# Supplementary material for: Psychological distress and its associated factors among cancer patients in Nepal: A cross-sectional study
Source: PLOS Ment Health. 2026 Mar 6;3(3):e0000419. doi: 10.1371/journal.pmen.0000419 (PMC12965590; doi:10.1371/journal.pmen.0000419)
Supplement: S1 Text — (PDF) [file pmen.0000419.s002.pdf]

S1 Text. Questionnaires used in the study

Section 1. Socio-demographic characteristics of the cancer patients (English and Nepali translation)		
**QUESTIONS**	**RESPONSES**	**Check the option/s**
Name of the health facility: स्वास्थ्य संस्थाको नाम		
	Bhaktapur Cancer Hospital (Government) भक्तपुर क्यान्सर अस्पताल	
	Nepal Cancer Hospital and Research Center (Private) नेपाल क्यान्सर अस्पताल	
Interview Place		
	IPD ward	
	Day care/ OPD ward	
	Others (Within hospital premise or others)	
Relationship with the patient उत्तरदाता बिरामीको को हो?		
	Husband/ Wife बुढा / बुढी	
	Son/ Daughter छोरा / छोरी	
	Father/ Mother बुवा / आमा	
	Relatives आफन्त	
	Friend/ Others साथीभाई / अन्य	
District (Permanent address) जिल्ला (स्थाई)		
Metro/ Sub-Metro/ Municipality/ Rural Municiaplity? महानगर / उपमहानगर / नगरपालिका / गाउँपालिका		
Ward number वार्ड नम्बर:		
Tole टोल		
Mobile Number मोवाईल नम्बर		
Interviewer's Initials अन्तर्वार्ताकर्ताको नाम		
Completed age (in years) पूरा भएको उमेर (वर्षमा)		
What is your sex? बिरामीको लिङ्ग		
	Male पुरुष	
	Female महिला	
What is your caste or ethnic composition? बिरामीको जात के हो?		
	Brahman ब्राह्मण	
	Chhetri क्षेत्रि	
	Janajati जनजाती	
	Others अन्य	
What is your marital status? बिरामीको वैवाहिक स्थिति ?		
	Single एकल	
	Married विवाहित	
	Divorced सम्बन्धविच्छेद भएको	
	Widowed विधवा	

	Others अन्य	
Number of years of education* NOTE: (*Class 10=10 years of education) (0 = No formal education; 99 = No formal education but able to read and write) बिरामीको शिक्षाको वर्ष संख्या * (*कक्षा १०=१० वर्षको शिक्षा) (० औपचारिक शिक्षा छैन; ९९ औपचारिक शिक्षा छैन तर पढ्न र लेख्न सक्षम छ)		
What was your occupation before diagnosis of disease? बिरामीको रोग लाग्नु पहिलेको पेशा के हो?		
	Farmer किसान	
	Labour श्रम / मजदुर	
	Government Employee सरकारी कर्मचारी	
	Private employee निजी कर्मचारी	
	Self-employed or Own business स्व-रोजगार / आफ्नै व्यवसाय	
	Job retirement नोकरी सेवानिवृत्ति	
	Student विद्यार्थी	
	Housewife/ Husband/ No professional work गृहिणी वा पति / कुनै व्यावसायिक काम नभएको	
	Others अन्य	
What is your current occupation? बिरामीको हालको पेशा के हो?		
	Farmer किसान	
	Labour श्रम / मजदुर	
	Government Employee सरकारी कर्मचारी	
	Private employee निजी कर्मचारी	
	Self-employed or Own business स्व-रोजगार / आफ्नै व्यवसाय	
	Job retirement नोकरी सेवानिवृत्ति	
	Student विद्यार्थी	
	Housewife/ Husband/ No professional work गृहिणी वा पति / कुनै व्यावसायिक काम नभएको	
	Others अन्य	
Number of family members who are economically active? बिरामीको परिवारमा कमाउने सदस्य सङ्ख्या कति छ ?		
Family's major occupation बिरामीको परिवारको प्रमुख पेशा		
	Farmer किसान	
	Labour श्रम / मजदुर	
	Government Employee सरकारी कर्मचारी	

	Private employee निजी कर्मचारी	
	Self-employed or Own business स्व-रोजगार / आफ्नै व्यवसाय	
	Job retirement नोकरी सेवानिवृत्ति	
	Student विद्यार्थी	
	Housewife/ Husband/ No professional work गृहिणी वा पति / कुनै व्यावसायिक काम नभएको	
	Others अन्य	
Number of Children in your family (less than 18 years)? १८ वर्षमुनिको सदस्य संख्या		
Number of Adults (18-64 years) in your family? १८-६४ वर्षबीचको सदस्य संख्या		
Number of old age members (65 years and above) in your family? ६५ वर्षमाथिको सदस्य संख्या		
How much is the household total monthly income? बिरामीको परिवारको कुल मासिक आय कति छ?		
How much is the household total monthly expenditure? बिरामीको घरको कुल मासिक खर्च कति छ?		
Are you insured under National Health Insurance Program of Nepal? (Social Health Insurance Program) के बिरामीको नेपालको राष्ट्रिय स्वास्थ्य बीमा कार्यक्रम अन्तर्गत बीमा भएको छ? (सामाजिक स्वास्थ्य बीमा कार्यक्रम)		
	Insured छ	
	Not Insured छैन	
Are you insured under any other insurance schemes? (e.g., Social Security Fund or Employment Provident Fund) बिरामीको अन्य कुनै बीमा योजना? (जस्तै, सामाजिक सुरक्षा कोष वा रोजगार सञ्चय कोष)		
	No छैन	
	Yes छ	
If Yes, what insurance? (e.g., Social Security Fund or Employment Provident Fund) छ भने कुन?		
Did you receive Rs. 1 Lakhs for cancer management through Bipanna Nagarik Kosh? विपन्न नागरिक कोषमार्फत क्यान्सर व्यवस्थापनका लागि रुपैयाँ एक लाख प्राप्त गर्नुभयो ?		
	No छैन	
	Yes छ	
Number of health facility visited for cancer management? बिरामीले क्यान्सर उपचारका क्रममा कति वटा स्वास्थ्य संस्था पुग्नुभयो ?		
**Section 2. Medical information of the cancer patients including clinical characteristics (English and Nepali translation)**		

Which type of cancer were you diagnosed with? बिरामीलाई कुन प्रकारको क्यान्सर भएको पत्ता लाग्यो?		
	Breast Cancer स्तन क्यान्सर	
	Cervical Cancer पाठेघरको मुखको क्यान्सर	
	Lung Cancer फोक्सोको क्यान्सर	
	Prostate Cancer प्रोस्टेट क्यान्सर	
	Other type of cancer अन्य	
What other type of cancer were you diagnosed with? बिरामीलाई कुन प्रकारको क्यान्सर भएको पत्ता लाग्यो?		
When was your case diagnosed? बिरामीको केस कहिले पत्ता लाग्यो?		
	More than 2 years दुइ वर्ष भन्दा बढी	
	1 year - 2 year १ देखि २ वर्ष	
	6 months - 1 year ६ महिना देखि १ वर्ष	
	Less than 6 months ६ महिनाभन्दा कम	
What was the stage of your Cancer at the time of diagnosis? पत्ता लागेको समयमा बिरामीको रोग कुन स्टेजमा थियो?		
	First stage पहिलो	
	Second stage दोस्रो	
	Third stage तेस्रो	
	Fourth stage चौथो	
When was the treatment protocol initiated? उपचार प्रोटोकल कहिले सुरु गरिएको थियो?		
	within 7 days of diagnosis पत्ता लागेको सात दिन भित्र	
	Within 1 month of diagnosis पत्ता लागेको महिना दिन भित्र	
	Within 3 months of diagnosis पत्ता लागेको ३ महिना दिन भित्र	
	Within 6 months of diagnosis पत्ता लागेको ६ महिना दिन भित्र	
	After 6 months of diagnosis पत्ता लागेको ६ महिना पछि	
	Not yet started सुरु गरेको छैन	
Which treatment did you receive so far? बिरामीले अहिलेसम्म केके उपचार पाउनुभयो?		
	Chemotherapy केमोथेरापी	
	Radiotherapy रेडियोथेरापी	
	Surgery शल्यक्रिया/ अप	
	Hormonal therapy हार्मोनल थेरापी	
	Supportive treatment सहायक उपचार	
	Others अन्य	

	None कुनै पनि छैन	
How many treatment cycles for chemotherapy did you complete? (In number) केमोथेरापीका लागि बिरामीले कति वटा उपचार चक्र पूरा गर्नुभयो? (संख्यामा)		
How many treatment cycles for radiotherapy did you complete? (In number) रेडियोथेरापीका लागि बिरामीले कति वटा उपचार चक्र पूरा गर्नुभयो? (संख्यामा)		
Are you suffering from any other chronic diseases? के बिरामी अन्य कुनै दिर्घ रोगबाट पीडित हुनुहुन्छ?		
	No छैन	
	Yes छ	
If yes, what chronic disease do you have besides cancer? कुन दिर्घ रोगबाट पीडित हुनुहुन्छ?		
Have you received OPD service/ Daycare within the last one year? गएको १ बर्षभित्र बिरामीले ओपिडि सेवा वा डे केयर सेवा लिनुभएको छ ?		
	No छैन	
	Yes छ	
**Section 3. OPD or ambulatory care expenses incurred during the cancer management (English and Nepali translation)**		
In the last 1 month (30 days), how many times did you visit OPD at oncology service providing health facilities for your cancer? विगत १ महिना (३० दिन) मा, बिरामीले क्यान्सरको लागि ओपिडी प्रदान गर्ने स्वास्थ्यसंस्थामा सेवा लिन कति पटक जानुभयो?		
Over the last 1 year (12 months), how many times did you visit OPD at oncology service providing health facilities for your cancer? विगत १ बर्ष (१२ महिना) मा, बिरामीले क्यान्सरको लागि ओपिडी प्रदान गर्ने स्वास्थ्यसंस्थामा सेवा लिन कति पटक जानुभयो?		
	Less than 10 times १० पटक भन्दा	
	10-20 times १० देखि २० पटक	
	More than 20 times २० पटकभन्दा बढी	
Consultation cost/ OPD Ticket cost in the last one month (30days)? परामर्श खर्च/ ओपीडी टिकेट खर्च (यो १ महिना भित्र)		
Consultation cost/ OPD Ticket cost in the last one year (12 months)? परामर्श खर्च/ ओपीडी टिकेट खर्च (बितेको १ वर्ष भित्र)		
Diagnostic tests/ Investigation/ Imaging cost (x-ray, MRI, etc.) in the last one month (30days)? डायग्नोस्टिक / अनुसन्धान / इमेजिंग (x-ray, MRI, etc.) खर्च (यो १ महिना भित्र)		
Diagnostic tests/ Investigation/ Imaging cost (x-ray, MRI, etc.) in the last one year (12 months)?		

डायग्नोस्टिक / अनुसन्धान / इमेजिंग (x-ray, MRI, etc.) खर्च (बितेको १ वर्ष भित्र)		
Cancer treatment cost in the last one month (30days)? क्यान्सरको उपचार खर्च (यो १ महिना भित्र)		
Cancer treatment cost in the last one year (12 months)? क्यान्सरको उपचार खर्च (बितेको १ वर्ष भित्र)		
Medicine and medical equipment cost in the last one month (30days)? औषधि र चिकित्सा उपकरणको खर्च (यो १ महिना भित्र)		
Medicine and medical equipment cost in the last one year (12 months)? औषधि र चिकित्सा उपकरणको खर्च (बितेको १ वर्ष भित्र)		
Therapeutic appliances and equipment cost in the last one month (30days)? थेराप्यूटिक सेवा तथा उपकरणसम्बन्धि खर्च (यो १ महिना भित्र)		
Therapeutic appliances and equipment cost in the last one year (12 months)? थेराप्यूटिक सेवा तथा उपकरणसम्बन्धि खर्च (बितेको १ वर्ष भित्र)		
Transportation cost in the last one month (30days)? यातायात खर्च (यो १ महिना भित्र)		
Transportation cost in the last one year (12 months)? यातायात खर्च (बितेको १ वर्ष भित्र)		
Food and other related cost in the last one month (30days)? खाना र अन्य सम्बन्धित खर्च (यो १ महिना भित्र)		
Food and other related cost in the last one year (12 months)? खाना र अन्य सम्बन्धित खर्च (बितेको १ वर्ष भित्र)		
How did you finance your OPD expense for cancer management? (Multiple choice) बिरामीले क्यान्सर व्यवस्थापनका लागि ओपिडी खर्च कसरी खर्च गर्नुभयो? (बहुविकल्पीय)		
	Current income of any household member/s or household income घरको कुनै पनि सदस्यको हालको आय वा घरको आय	
	Savings of any household member/s or household savings कुनै पनि परिवारका सदस्यको बचत वा घरको बचत	
	Sold items (land, property, livestock, jewelry) or pawning assets (Dharauti) बेचेका वस्तुहरू (जमिन, सम्पत्ति, पशुधन, गहना) वा बन्धक राख्ने सम्पत्ति (धरौटि)	
	Ask relatives and friends for financial support आफन्त र साथीभाइबाट आर्थिक सहयोग	

	Borrow from financial institutions (Banks, microfinance schemes) वित्तीय संस्थाहरूबाट ऋण (बैंक, माइक्रोफाइनान्स योजनाहरू)	
	Received subsidy from government/NGOs/charities सरकार/गैरसरकारी संस्था/परोपकारी संस्थाहरूबाट अनुदान प्राप्त	
	Formal insurance औपचारिक बीमा	
	Cut down on food and other HH consumption खाना र अन्य घरेलु खपतमा कटौती	
	Cut down on non-food expenditure गैर-खाद्य खर्च कटौती	
	Others अन्य	
What type of subsidy? कस्तो अनुदान ?		
Amount of Subsidy? अनुदानको रकम ?		
Cut down on which non-food expenditure? कुन गैर-खाद्य खर्चमा कटौती?		
	Reduced educational expenditure (e.g., compromised child education) शैक्षिक खर्चमा कमी (उदाहरणका लागि, बाल शिक्षामा सम्झौता)	
	Reduce Medical visits/treatment चिकित्सा भ्रमण/उपचार घटाएको	
	Cut down on other non-food expenditures अन्य गैर-खाद्य खर्चमा कटौती	
	Cut down on what other non-food expenditures? अन्य कुन गैर-खाद्य खर्चमा कटौती?	
How did you finance in other ways? बिरामीले क्यान्सर व्यवस्थापनका लागि आइपिडी खर्च अन्य कुन तरिकाबाट जुटाउनुभयो ?		
During the last 12 months, how much did you spend for traditional treatment? विगत १२ महिनामा बिरामीले परम्परागत उपचारमा कति खर्च गर्नुभयो?		
Have you received IPD service within the last one year? गएको १ बर्षभित्र बिरामीले आईपिडि (भर्ना भएर) सेवा लिनुभएको छ ?		
	No छैन	
	Yes छ	
**Section 4. IPD care expenses incurred during the cancer management (English and Nepali translation)**		
In the last 1 month (30 days), how many times did you visit IPD at oncology service providing		

health facilities for your cancer? विगत १ महिना (३० दिन) मा, बिरामी क्यान्सरको लागि आईपीडी प्रदान गर्ने स्वास्थ्यसंस्थामा सेवा लिन कति पटक जानुभयो?	
How many days did you spend in the IPD ward in the last 30 days? (Duration of Stay) विगत १ महिना (३० दिन) मा, बिरामी क्यान्सरको लागि आईपीडी प्रदान गर्ने स्वास्थ्यसंस्थामा कति दिन बिताउनुभयो?	
In the last 1 year (12 months), how many times did you visit IPD at oncology service providing health facilities for your cancer? विगत १ बर्ष (१२ महिना) मा, बिरामी क्यान्सरको लागि आईपीडी प्रदान गर्ने स्वास्थ्यसंस्थामा सेवा लिन कति पटक जानुभयो?	
Over the last 1 year (12 months), how many days did you stay at IPD ward at oncology service providing health facilities for your cancer management? (Duration of Stay) विगत १ वर्ष (१२ महिना) मा, बिरामी क्यान्सरको लागि आईपीडी प्रदान गर्ने स्वास्थ्यसंस्थामा कति दिन बिताउनुभयो?	
Consultation cost (including Round Fees) in the last one year (12 months)? परामर्श खर्च + राउन्ड फि (यो १ वर्ष भित्र)	
Inpatient admission cost in the last one month (30 days)? इनपेशेंट एडमिशन खर्च (यो १ महिना भित्र)	
Inpatient admission cost in the last one year (12 months)? इनपेशेंट एडमिशन खर्च (यो १ वर्ष भित्र)	
Bed charge in the last one month (30days)? बेड चार्ज (यो १ महिना भित्र)	
Bed charge in the last one year (12 months)? बेड चार्ज (यो १ बर्ष भित्र)	
Diagnostic tests/ Investigation/ Imaging cost (x-ray, MRI, etc.) in the last one month (30days)? डायग्नोस्टिक / जाँच / इमेजिंग (x-ray, MRI, etc.) खर्च (यो १ महिना भित्र)	
Diagnostic tests/ Investigation/ Imaging cost (x-ray, MRI, etc.) in the last one year (12 months)? डायग्नोस्टिक / अनुसन्धान / इमेजिंग खर्च (x-ray, MRI, etc.) (यो १ वर्ष भित्र)	
Cancer treatment cost in the last one month (30days)? क्यान्सरको उपचार खर्च (यो १ महिना भित्र)	
Cancer treatment cost in the last one year (12 months)? क्यान्सरको उपचार खर्च (यो १ वर्ष भित्र)	
Medicine and medical equipment cost in the last one month (30days)? औषधि र चिकित्सा उपकरणको खर्च (यो १ महिना भित्र)	

Medicine and medical equipment cost in the last one year (12 months)? औषधि र चिकित्सा उपकरणको खर्च (यो १ वर्ष भित्र)		
Therapeutic appliances and equipment cost in the last one month (30days)? थेराप्यूटिक सेवा तथा उपकरणसम्बन्धि खर्च (यो १ महिना भित्र)		
Therapeutic appliances and equipment cost in the last one year (12 months)? थेराप्यूटिक सेवा तथा उपकरणसम्बन्धि खर्च (यो १ वर्ष भित्र)		
Transportation cost in the last one month (30days)? यातायात खर्च (यो १ महिना भित्र)		
Transportation cost in the last one year (12 months)? यातायात खर्च (यो १ वर्ष भित्र)		
Food and other related cost in the last one month (30days)? खाना र अन्य सम्बन्धित खर्च (यो १ महिना भित्र)		
Food and other related cost in the last one year (12 months)? खाना र अन्य सम्बन्धित खर्च (यो १ वर्ष भित्र)		
How did you finance your IPD expense for cancer management? (Multiple choice) क्यान्सर व्यवस्थापनका लागि आइपिडी खर्च कसरी जुटाउनु भयो? (बहुविकल्पीय)		
	Current income of any household member/s or household income घरको कुनै पनि सदस्यको हालको आय वा घरको आय	
	Savings of any household member/s or household savings कुनै पनि परिवारका सदस्यको बचत वा घरको बचत	
	Sold items (land, property, livestock, jewelry) or pawning assets (Dharauti) बेचेका वस्तुहरू (जमिन, सम्पत्ति, पशुधन, गहना) वा बन्धक राख्ने सम्पत्ति (धरौटि)	
	Ask relatives and friends for financial support आफन्त र साथीभाइबाट आर्थिक सहयोग	
	Borrow from financial institutions (Banks, microfinance schemes) वित्तीय संस्थाहरूबाट ऋण (बैंक, माइक्रोफाइनान्स योजनाहरू)	
	Received subsidy from government/NGOs/charities सरकार/गैरसरकारी संस्था/परोपकारी संस्थाहरूबाट अनुदान प्राप्त भएको	
	Formal insurance औपचारिक बीमा	

	Cut down on food and other HH consumption खाना र अन्य एचएच खपतमा कटौती	
	Cut down on non-food expenditure गैर-खाद्य खर्चमा कटौती	
	Others अन्य	
What type of subsidy? कस्तो अनुदान ?		
Amount of Subsidy? अनुदानको रकम ?		
Cut down on which non-food expenditure? कुन गैर-खाद्य खर्चमा कटौती?		
	Reduced educational expenditure (e.g., compromised child education) शैक्षिक खर्चमा कमी (उदाहरणका लागि, बाल शिक्षामा सम्झौता)	
	Reduce Medical visits/treatment चिकित्सा भ्रमण/उपचार घटाएको	
	Cut down on other non-food expenditures अन्य गैर-खाद्य खर्चमा कटौती	
	Cut down on what other non-food expenditures? अन्य कुन गैर-खाद्य खर्चमा कटौती?	
How did you finance in other ways? क्यान्सर व्यवस्थापनका लागि आइपिडी खर्च अन्य कुन तरिकाबाट जुटाउनुभयो ?		

Section 5. Patients' household essential consumptions based on COICOP 2018 categories (English and Nepali translation)

	**Expenses before onset of cancer (NRs.) क्यान्सर लग्नु पहिलाको खर्च (रु.)**	**Expenses after onset of cancer (NRs.) क्यान्सर लागिसकेपछिको खर्च (रु.)**
**खाद्य र गैर-अल्कोहलयुक्त पेय पदार्थहरू Food and non-alcoholic beverages**		
प्रति हप्ता per week		
प्रति महिना per month		
प्रति बर्ष per year		
**अल्कोहलयुक्त पेय पदार्थ र सुर्तीजन्य पदार्थ Alcoholic beverages and tobacco**		
प्रति हप्ता per week		
प्रति महिना per month		
प्रति बर्ष per year		
**लुगाफाटा र जुत्ता Clothing and footwear**		
प्रति महिना per month		
प्रति बर्ष per year		
**आवास, पानी, बिजुली, ग्याँस र अन्य ईन्धन Housing, water, gas, electricity and other fuels**		
प्रति महिना per month		

प्रति बर्ष per year		
**फर्निसिङ, घरायसी उपकरण र घरको नियमित मर्मतसम्भार Furnishings, household equipment and routine maintenance of the house**		
प्रति महिना per month		
प्रति बर्ष per year		
**स्वास्थ्य Health**		
प्रति महिना per month		
प्रति बर्ष per year		
**यातायात Transportation**		
प्रति महिना per month		
प्रति बर्ष per year		
**सञ्चार Communication**		
प्रति महिना per month		
प्रति बर्ष per year		
**मनोरञ्जन र संस्कृति Recreation and culture**		
प्रति महिना per month		
प्रति बर्ष per year		
**शिक्षा Education**		
प्रति महिना per month		
प्रति बर्ष per year		
**रेस्टुरेन्ट र होटल Restaurants and hotels**		
प्रति महिना per month		
प्रति बर्ष per year		
**विविध वस्तु र सेवाहरू Miscellaneous goods and services**		
प्रति महिना per month		
प्रति बर्ष per year		

Section 6. Socio-Demographics of Caregivers of Cancer Patients (English and Nepali translation)

Completed age (in years) पूरा भएको उमेर (वर्षमा)		
What is your sex? लिङ्ग		
Male पुरुष		
Female महिला		
What is your caste or ethnic compostion? तपाईंको जात के हो?		
	Brahman ब्राह्मण	
	Chhetri क्षेत्रि	
	Janajati जनजाती	
	Others अन्य	
What is your marital status? तपाईंको वैवाहिक स्थिति ?		
	Single एकल	
	Married विवाहित	

	Divorced सम्बन्धविच्छेद भएको	
	Widowed विधवा	
	Others अन्य	
Number of years of education* NOTE: (*Class 10=10 years of education) (0 = No formal education; 99 = No formal education but able to read and write) शिक्षाको वर्ष संख्या * (*कक्षा १०=१० वर्षको शिक्षा) (० औपचारिक शिक्षा छैन; ९९ औपचारिक शिक्षा छैन तर पढ्न र लेख्न सक्षम छ)		
What is your current occupation? तपाईंको हालको पेशा के हो?		
	Farmer किसान	
	Labour श्रम / मजदुर	
	Government Employee सरकारी कर्मचारी	
	Private employee निजी कर्मचारी	
	Self-employed or Own business स्व-रोजगार / आफ्नै व्यवसाय	
	Job retirement नोकरी सेवानिवृत्ति	
	Student विद्यार्थी	
	Housewife/ Husband/ No professional work गृहिणी वा पति / कुनै व्यावसायिक काम नभएको	
	Others अन्य	
Relationship with the patient विरामीको तपाई को हो?		
	Husband/ Wife बुढा / बुढी	
	Son/ Daughter छोरा / छोरी	
	Father/ Mother बुवा / आमा	
	Relatives आफन्त	
	Friend/ Others साथीभाई / अन्य	
Living with patient? बिरामीसंगै बस्ने हो ?		
	No हैन	
	Yes हो	
Household size? परिवार सदस्य संख्या		
How much is your monthly income? तपाईको कुल मासिक आय कति छ?		
Number of health facility visited for cancer management of your family member as a caregiver बिरामीको क्यान्सर उपचारका क्रममा तपाई कति वटा स्वास्थ्य संस्था पुग्नुभयो?		
Care taken in terms of? कुन कुन तरिकाले हेरचाह गरिरहनु भएको छ ?		
	Domestic help घरेलु काममा सहयोग गरेर	

	Financial help आर्थिक तरिकाले	
	Help in transportation to health facilities अस्पताल ल्याउने लैजाने स्वास्थ्य (यातायात)	
	Other अन्य	
Duration of care work? हेरचाह गर्न थालेको कति समय भयो?		
	Less than 3 months ३ महिना भन्दा कम	
	3-6 months ३-६ महिना	
	6 months - 1year ६ महिना - १ बर्ष	
	1-2 years १-२ बर्ष	
	More than 2 years २ बर्ष भन्दा बढी	
Hours of care per day दिनमा कति घण्टा हेरचाह गर्नुहुन्छ?		
	Less than 5 hours ५ घण्टा भन्दा कम	
	5-10 hours ५-१० घण्टा	
	More than 10 hours १० घण्टा भन्दा बढी	

Section 7. Validated Nepali version of the DASS-21 tool (originally developed by Lovibond and Lovibond to assess psychological distress along three dimensions: depression, anxiety, and stress)

**Over the past week.... गत हप्ताभरि.....**	**हेरचाहकर्ताको मानसिक स्वास्थ्य psychological health of caregivers**	**बिरामीको मानसिक स्वास्थ्य psychological health of cancer patients**
**मलाई तनावमुक्त हुन गाह्रो लाग्यो । यस्तो कत्तिको भयो ?**		
पटक्कै भएन		
कुनै कुनै समय भयो		
धेरै समयसम्म भयो		
पूर्णरूपमा वा प्रायजसो समयमा भयो		
**मेरो मुख सुख्खा भएको मलाई थाहा थियो । यस्तो कत्तिको भयो ?**		
पटक्कै भएन		
कुनै कुनै समय भयो		
धेरै समयसम्म भयो		
पूर्णरूपमा वा प्रायजसो समयमा भयो		
**मैले कुनै पनि सकारात्मक भावनाको अनुभव गर्न साकिन । यस्तो कत्तिको भयो ?**		
पटक्कै भएन		
कुनै कुनै समय भयो		
धेरै समयसम्म भयो		
पूर्णरूपमा वा प्रायजसो समयमा भयो		
**मलाई सास फेर्न गाह्रो भएको मैले अनुभव गरे (उदाहरण दम बढ्नु, सास रोकिनु) । यस्तो कत्तिको भयो ?**		
पटक्कै भएन		
कुनै कुनै समय भयो		

धेरै समयसम्म भयो		
पूर्णरूपमा वा प्रायजसो समयमा भयो		
**मैले कुनै पनि नयाँ काम आफै सुरु गर्न गाह्रो परेको अनुभव गरे। यस्तो कत्तिको भयो ?**		
पटक्कै भएन		
कुनै कुनै समय भयो		
धेरै समयसम्म भयो		
पूर्णरूपमा वा प्रायजसो समयमा भयो		
**कतिपय स्थितिहरूमा मैले चाहिनेभन्दा बढी प्रतिकार गरे। यस्तो कत्तिको भयो ?**		
पटक्कै भएन		
कुनै कुनै समय भयो		
धेरै समयसम्म भयो		
पूर्णरूपमा वा प्रायजसो समयमा भयो		
**मैले आफू काँपेको अनुभव गरे (उदाहरण हातहरू)। यस्तो कत्तिको भयो ?**		
पटक्कै भएन		
कुनै कुनै समय भयो		
धेरै समयसम्म भयो		
पूर्णरूपमा वा प्रायजसो समयमा भयो		
**म धेरै अतालिएको मलाई अनुभव भयो। यस्तो कत्तिको भयो ?**		
पटक्कै भएन		
कुनै कुनै समय भयो		
धेरै समयसम्म भयो		
पूर्णरूपमा वा प्रायजसो समयमा भयो		
**म डराउने अनि आफैलाई मूर्ख ठान्ने परिस्थितिहरूको विषयमा चिन्तित भएँ। यस्तो कत्तिको भयो ?**		
पटक्कै भएन		
कुनै कुनै समय भयो		
धेरै समयसम्म भयो		
पूर्णरूपमा वा प्रायजसो समयमा भयो		
**मैले आशावादी हुनुपर्ने केही कारण देखिन। यस्तो कत्तिको भयो ?**		
पटक्कै भएन		
कुनै कुनै समय भयो		
धेरै समयसम्म भयो		
पूर्णरूपमा वा प्रायजसो समयमा भयो		
**मैले आफैंलाई अशान्त भएको महशुस गरे। यस्तो कत्तिको भयो ?**		
पटक्कै भएन		
कुनै कुनै समय भयो		
धेरै समयसम्म भयो		
पूर्णरूपमा वा प्रायजसो समयमा भयो		
**मैले आराम गर्न गाह्रो भएको महशुस गरे। यस्तो कत्तिको भयो ?**		
पटक्कै भएन		
कुनै कुनै समय भयो		
धेरै समयसम्म भयो		
पूर्णरूपमा वा प्रायजसो समयमा भयो		
**म आफू दुःखी र उदास भएको महसुस गरे। यस्तो कत्तिको भयो ?**		
पटक्कै भएन		
कुनै कुनै समय भयो		

धेरै समयसम्म भयो		
पूर्णरूपमा वा प्रायजसो समयमा भयो		
**मैले गर्दै गरेको कुरामा बाधा पर्दा मलाई हतास लाग्ने गर्थ्यो। यस्तो कत्तिको भयो ?**		
पटक्कै भएन		
कुनै कुनै समय भयो		
धेरै समयसम्म भयो		
पूर्णरूपमा वा प्रायजसो समयमा भयो		
**मैले डराउन लागेको वा आतंकित भएको महसुस गरे। यस्तो कत्तिको भयो ? यस्तो कत्तिको भयो ?**		
पटक्कै भएन		
कुनै कुनै समय भयो		
धेरै समयसम्म भयो		
पूर्णरूपमा वा प्रायजसो समयमा भयो		
**म कुनै पनि कुरोको विषयमा उत्साहित बन्न असक्षम भएँ। यस्तो कत्तिको भयो ?**		
पटक्कै भएन		
कुनै कुनै समय भयो		
धेरै समयसम्म भयो		
पूर्णरूपमा वा प्रायजसो समयमा भयो		
**म एउटा नालायक व्यक्ति रहेछु जस्तो मलाई लाग्यो। यस्तो कत्तिको भयो ?**		
पटक्कै भएन		
कुनै कुनै समय भयो		
धेरै समयसम्म भयो		
पूर्णरूपमा वा प्रायजसो समयमा भयो		
**म भावुक भएको जस्तो मलाई महसुस भयो। यस्तो कत्तिको भयो ?**		
पटक्कै भएन		
कुनै कुनै समय भयो		
धेरै समयसम्म भयो		
पूर्णरूपमा वा प्रायजसो समयमा भयो		
**शरीरिक परिश्रमविना नै मेरो मुट्टूको धड्कन (हृदयगति) बढेको महसुस गरे। यस्तो कत्तिको भयो ?**		
पटक्कै भएन		
कुनै कुनै समय भयो		
धेरै समयसम्म भयो		
पूर्णरूपमा वा प्रायजसो समयमा भयो		
**विना कुनै कारण मैले डराएको अनुभव गरे। यस्तो कत्तिको भयो ?**		
पटक्कै भएन		
कुनै कुनै समय भयो		
धेरै समयसम्म भयो		
पूर्णरूपमा वा प्रायजसो समयमा भयो		
**मलाई जीवन अर्थहीन भएको जस्तो लाग्यो। यस्तो कत्तिको भयो ?**		
पटक्कै भएन		
कुनै कुनै समय भयो		
धेरै समयसम्म भयो		
पूर्णरूपमा वा प्रायजसो समयमा भयो		

***
