# Supplementary material for: Psychological distress and its associated factors among cancer patients in Nepal: A cross-sectional study
Source: PLOS Ment Health. 2026 Mar 6;3(3):e0000419. doi: 10.1371/journal.pmen.0000419 (PMC12965590; doi:10.1371/journal.pmen.0000419)
Supplement: S2 Text — (DOCX) [file pmen.0000419.s003.docx]

**Sampling frame, sample size, and sampling procedure**

A total of 400 cancer patients were recruited from selected tertiary-level hospitals in Nepal as part of a larger study investigating the economic burden and psychological impacts of cancer. The initial sample size was calculated using the formula for comparing two independent groups of equal sizes (1):

$$n=\frac{2\left( Z_{\alpha}+Z_{\beta} \right)}{\left( \delta/\sigma\right)^{2}}$$

where:

δ = |μ_0_ − μ_1_| is the smallest difference between the means of two groups that the statistical test can detect

σ: population variance

Z_α_: standardized value associated with α, the level of significance

Z_β_: standardized value associated with β, the type II error.

For α = 0.05, Z_α_ = 1.96

Since power was set at 80% (β = 0.20); for β = 0.20, Z_β_ =0.84

M (multiplier) = 2 (Z_α_+Z_β_)^2^= 2(1.96+0.84)^2^ = 15.68 ≈ 16

$$n=\frac{M}{\Delta^{2}}$$

Where: ∆ = |μ_0_−μ_1_| / σ = δ / σ

Δ is the standardized difference between means. Assuming a smallest detectable standardized mean difference of ∆ = 0.20,

n per group = 16/∆^2^ = 16/ (0.2)^2^ = 200.

Therefore, a total of 400 samples were needed and collected.

At each site, participants were selected using a two-stage random sampling approach. First, a sampling frame of all operational dates within the study period was created: 51 consecutive dates from 28 March to 17 May 2023 for BCH, and 28 dates from 18 April to 15 May 2023 for NCHRC. From these frames, 17 and 13 dates, respectively, were randomly selected without replacement using the sample() function in R (seed = 1 for BCH, seed = 2 for NCHRC) (S1 and S2 Tables). On the selected dates, all eligible cancer patients attending outpatient, inpatient, or daycare services between 08:00 and 18:00 were invited to participate, in collaboration with the nurses in charge, ensuring that patient inclusion did not depend on staff discretion. Weekends and public holidays were included to capture the full spectrum of patient visits.

For this specific analysis focused on psychological distress, no stratification by facility type was performed. Of the 400 participants enrolled, 262 consented to and completed the Depression, Anxiety and Stress Scale - 21 Items (DASS-21) assessment tool for measuring psychological distress. The exclusion of non-respondents may introduce non-response bias, which is acknowledged as a limitation. Analyses of economic outcomes from the broader study, using the same sample, will be reported separately (2).

**References**

1. Stephen B. Hulley SRC, Warren S. Browner, Deborah G. Grady, Thomas B. Newm. Designing Clinical Research. 2001.

2. [Author's own], et al (forthcoming) Healthcare expenses, financial coping strategies and their impact on household welfare among cancer patients in public and private hospitals in Nepal: A cross-sectional study.
