# Supplementary material for: Psychological distress and its associated factors among cancer patients in Nepal: A cross-sectional study
Source: PLOS Ment Health. 2026 Mar 6;3(3):e0000419. doi: 10.1371/journal.pmen.0000419 (PMC12965590; doi:10.1371/journal.pmen.0000419)
Supplement: S3 Text — (DOCX) [file pmen.0000419.s004.docx]

**S3 Text. Operational definitions and scoring criteria of the DASS-21 instrument**

**DASS-21:** Refers to depression, anxiety, and stress scale, which consists of 21 items.

***Level of depression:*** Level of symptoms of depression distinguished by the DASS-21 scale. These are categorized as Normal (0–9), mild (10–13), moderate depression (14–20), severe depression (21–27) and extremely severe depression (>27). For logistic regression analysis, these have been categorized as No Depression (0–9) and Depression (>9).

***Level of anxiety:*** Level of symptoms of anxiety distinguished by the DASS-21 scale. These are categorized as Normal (0–7), mild (8–9), moderate anxiety (10–14), severe anxiety (15–19), and extremely severe anxiety (>19). For logistic regression analysis, these have been categorized as No anxiety (0–7) and Anxiety (>7).

***Level of stress***: Level of symptoms of stress distinguished by the DASS-21 scale. These are categorized as Normal (0–14), mild (15–18), moderate stress (19–25), severe stress (26–33), and extremely severe stress (>33). For logistic regression analysis, these have been categorized as No stress (0–14) and Stress (>14).

***Cut-off scores:*** For logistic regression analysis, participants with depression scores >9, anxiety scores >7, and stress scores >14 were categorized as having depression, anxiety, and stress, respectively. These cutoffs, based on established DASS-21 scoring guidelines, allow for meaningful identification of participants at risk for clinically relevant psychological distress (1).
