# Supplementary material for: Psychological distress and its associated factors among cancer patients in Nepal: A cross-sectional study
Source: PLOS Ment Health. 2026 Mar 6;3(3):e0000419. doi: 10.1371/journal.pmen.0000419 (PMC12965590; doi:10.1371/journal.pmen.0000419)
Supplement: S1 Text — (PDF) [file pmen.0000419.s005.pdf]

अध्ययन सारांश

पृष्ठभूमि

क्यान्सर व्यवस्थापनको क्रममा विभिन्न जटिल उपचार प्रक्रियाहरूलाई प्राथमिकतामा राख्नुपर्ने भएकाले क्यान्सर रोगीहरूमा हुनसक्ने मनोवैज्ञानिक तनावलाई त्यति गम्भीरतापूर्वक हेरिँदैन। यसले क्यान्सर व्यवस्थापनको क्रममा हुने मनोवैज्ञानिक तनावको जोखिम अझ बढाउन सक्छ, जसले गर्दा क्यान्सर रोगीहरुको स्वास्थ्य र जीवनको गुणस्तरमा नकारात्मक असर पर्छ। यस अध्ययनले मनोवैज्ञानिक तनाव भएका क्यान्सर रोगीहरूको अनुपात र यससँग सम्बन्धित कारकको मुल्यांकन गर्ने लक्ष्य राखेको छ।

अध्ययन विधि

नेपालका दुई ठूला रेफरल स्तरका अस्पतालहरूमा क्यान्सर बिरामीहरूमाझ गरिएको यस अध्ययनका लागि Lovibond and Lovibond (1995) द्वारा विकसित DASS-21 उपकरणको मान्य नेपाली संस्करण प्रयोग गरिएको थियो। यसको प्रयोगबाट महत्वपूर्ण तीन मनोवैज्ञानिक आयामहरू- डिप्रेसन, एन्जाईटि र स्ट्रेसलाई मूल्याङ्कन गरियो। यस अध्ययनमा कुल २६२ क्यान्सर बिरामीहरुले सहभागिता जनाएका थिए। डेटा क्लिनिङ, कोडिङ र विश्लेषण गर्न SPSS Version 27 प्रयोग गरिएको थियो। परिणामको विश्लेषणका लागि ०.०५ भन्दा कम P-value लाई सांख्यिकीय रूपमा महत्वपूर्ण सम्बन्धहरूको संकेत गर्न प्रयोग गरियो। यसैगरी, यस अध्ययनमा बाइनरी लजिस्टिक रिग्रेसन मोडेल प्रयोग गरेर Odds Ratio गणना गरिएको थियो, र Multivariate लजिस्टिक रिग्रेसन मोडेलमा आवश्यक समायोजन गरिएको थियो।

परिणाम

क्यान्सर रोगीहरूको दुई तिहाइभन्दा बढीमा डिप्रेसन (६६.८%) र एन्जाईटि (६८.७%) का लक्षणहरू देखिए, र लगभग हरेक पाँच जनामध्य तीन जनामा स्ट्रेसका लक्षणहरू (५८.०%) देखिए। वृद्ध, बेरोजगार, न्यून शैक्षिकस्तर भएका र उच्च चरणको क्यान्सर भएकाहरूलाई तनाव हुने जोखिम बढी देखियो। पहिलो चरणको क्यान्सर भएको पत्ता लागेकाहरूलाई सन्दर्भमा राखेर हेर्ने हो भने, दोस्रो, तेस्रो र चौथो चरणको क्यान्सर भएको पत्ता लागेकाहरूमा डिप्रेसनका लक्षणहरू हुने सम्भाव्यता अनुपात (odds) क्रमशः २.७५ बढी (cOR २.७५; ९५% CI १.३९, ५.४२; p-value = ०.००४), २.८१ बढी (cOR २.८१; ९५% CI १.४०, ५.६४; p-value = ०.००४) र ४.२१ बढी (cOR ४.२१; ९५% CI १.३९, १२.७६; p-value = ०.०११) रहेको पाईयो। क्यान्सरका बिरामीहरूको परिवारले आफ्नै खल्तीबाट खर्च (Out-of-pocket expenses), विनाशकारी स्वास्थ्य खर्च (Catastrophic health expenses) तथा क्यान्सर लागेकै कारणले गरिबी (Impoverishment) बेहोर्नु परे तापनि, यी सूचकहरू र मनोवैज्ञानिक तनावबीच सांख्यिकीय रूपमा महत्वपूर्ण सम्बन्ध भेटिएन। यसले के संकेत गर्छ भने, क्यान्सर रोगीहरुमा हुने मनोवैज्ञानिक तनाव आर्थिक भारको कारणले मात्र नभई मुख्य रूपमा रोग, रोगको व्यवस्थापनका जटिलता, वा अन्य सामाजिक-सांस्कृतिक कारण मात्र नभई यस अध्ययनका कमी-कमजोरीहरुले गर्दा उत्पन्न भएको हुन सक्छ।

अध्ययनको सार

नेपालमा क्यान्सरका बिरामीहरूको जीवनस्तर सुधार गर्न सरकारले हालै जारी गरेको क्यान्सर नियन्त्रण रणनीतिलाई कडाईका साथ कार्यान्वयन र अनुगमन गर्नुपर्छ। क्यान्सर रोगका कारण मानसिक स्वास्थ्यमा पर्ने प्रभावसँग जुध्न अस्पताल र समुदायमा आधारित स्क्रिनिङ तथा परामर्श सेवाहरू तत्काल आवश्यक भएका छन्।

मुख्य शब्दहरु: क्यान्सर, क्यान्सर बिरामीहरु, मानसिक स्वास्थ्य, मनोवैज्ञानिक तनाव।
