# Supplementary material for: Psychological distress and its associated factors among cancer patients in Nepal: A cross-sectional study
Source: PLOS Ment Health. 2026 Mar 6;3(3):e0000419. doi: 10.1371/journal.pmen.0000419 (PMC12965590; doi:10.1371/journal.pmen.0000419)
Supplement: S1 Table — (PDF) [file pmen.0000419.s006.pdf]

S1 Table. Simple random sampling for public hospital

S.N.	Dates	Simple Random Sampling of the Dates
1	28-Mar-23	
2	29-Mar-23	
3	30-Mar-23	
4	31-Mar-23	
5	1-Apr-23	
6	2-Apr-23	
7	3-Apr-23	
8	4-Apr-23	
9	5-Apr-23	
10	6-Apr-23	
11	7-Apr-23	
12	8-Apr-23	
13	9-Apr-23	
14	10-Apr-23	
15	11-Apr-23	
16	12-Apr-23	
17	13-Apr-23	
18	14-Apr-23	
19	15-Apr-23	
20	16-Apr-23	
21	17-Apr-23	
22	18-Apr-23	
23	19-Apr-23	
24	20-Apr-23	
25	21-Apr-23	
26	22-Apr-23	
27	23-Apr-23	
28	24-Apr-23	
29	25-Apr-23	

30	26-Apr-23	
31	27-Apr-23	
32	28-Apr-23	
33	29-Apr-23	
34	30-Apr-23	
35	1-May-23	
36	2-May-23	
37	3-May-23	
38	4-May-23	
39	5-May-23	
40	6-May-23	
41	7-May-23	
42	8-May-23	
43	9-May-23	
44	10-May-23	
45	11-May-23	
46	12-May-23	
47	13-May-23	
48	14-May-23	
49	15-May-23	
50	16-May-23	
51	17-May-23	

Note: The sample() function in R was employed to generate random numbers.
