# Supplementary material for: Psychological distress and its associated factors among cancer patients in Nepal: A cross-sectional study
Source: PLOS Ment Health. 2026 Mar 6;3(3):e0000419. doi: 10.1371/journal.pmen.0000419 (PMC12965590; doi:10.1371/journal.pmen.0000419)
Supplement: S2 Table — (PDF) [file pmen.0000419.s007.pdf]

**S2 Table. Simple random sampling for private hospital**

| S.N. | Dates | Simple Random Sampling of the Dates |
|---|---|---|
| 1 | 18-Apr-23 | |
| 2 | 19-Apr-23 | |
| 3 | 20-Apr-23 | |
| 4 | 21-Apr-23 | |
| 5 | 22-Apr-23 | |
| 6 | 23-Apr-23 | |
| 7 | 24-Apr-23 | |
| 8 | 25-Apr-23 | |
| 9 | 26-Apr-23 | |
| 10 | 27-Apr-23 | |
| 11 | 28-Apr-23 | |
| 12 | 29-Apr-23 | |
| 13 | 30-Apr-23 | |
| 14 | 1-May-23 | |
| 15 | 2-May-23 | |
| 16 | 3-May-23 | |
| 17 | 4-May-23 | |
| 18 | 5-May-23 | |
| 19 | 6-May-23 | |
| 20 | 7-May-23 | |
| 21 | 8-May-23 | |
| 22 | 9-May-23 | |
| 23 | 10-May-23 | |
| 24 | 11-May-23 | |
| 25 | 12-May-23 | |
| 26 | 13-May-23 | |
| 27 | 14-May-23 | |
| 28 | 15-May-23 | |

Note: The sample() function in R was employed to generate random numbers.
