# Supplementary material for: Psychological distress and its associated factors among cancer patients in Nepal: A cross-sectional study
Source: PLOS Ment Health. 2026 Mar 6;3(3):e0000419. doi: 10.1371/journal.pmen.0000419 (PMC12965590; doi:10.1371/journal.pmen.0000419)
Supplement: S3 Table — (DOCX) [file pmen.0000419.s008.docx]

**S3 Table. Bivariate and multivariate for socio-demographic and clinical characteristic variables with anxiety symptoms**

| **Patients’ characteristics** | **Anxiety symptoms** | | **Model I,** | **p-value** | **Model II,** | **p-value** |
| --- | --- | --- | --- | --- | --- | --- |
|  | **Yes** | **No** | **cOR (95%CI)** |  | **aOR (95%CI)** |  |
|  | **n (%)** | **n (%)** |  |  |  |  |
| **Age (in years)** Mean (SD): 53.61 (14.41) | | | | | | |
| Less than 40 (50) | 29 (58.0) | 21 (42.0) | Ref | Ref | Ref | Ref |
| 40-59 (126) | 87 (69.0) | 39 (31.0) | 1.62 (0.82, 3.18) | 0.165 | 1.00 (0.42, 2.42) | 0.999 |
| 60 and above (86) | 64 (74.4) | 22 (25.6) | **2.11 (1.00, 4.42)** | **0.049** | 0.88 (0.33, 2.38) | 0.804 |
| **Sex** | | | | | | |
| Male (97) | 65 (67.0) | 32 (33.0) | Ref | Ref |  |  |
| Female (165) | 115 (69.7) | 50 (30.3) | 1.13 (0.66, 1.94) | 0.651 |  |  |
| **Marital status** | | | | | | |
| Single (25) | 14 (56.0) | 11 (44.0) | Ref | Ref |  |  |
| Married (218) | 151 (69.3) | 67 (30.7) | 1.77 (0.76, 4.10) | 0.183 |  |  |
| Widowed (19) | 15 (78.9) | 4 (21.1) | 2.95 (0.76, 11.44) | 0.118 |  |  |
| **Occupation status after cancer diagnosis** | | | | | | |
| Employed (61) | 35 (57.4) | 26 (42.6) | Ref | Ref | Ref | Ref |
| Unemployed (201) | 145 (72.1) | 56 (27.9) | **1.92 (1.06, 3.48)** | **0.031** | 1.22 (0.59, 2.51) | 0.590 |
| **Level of education** | | | | | | |
| Up to 5 years (131) | 101 (77.1) | 30 (22.9) | **4.12 (1.96, 8.66)** | **<0.001** | **4.42 (1.71, 11.47)** | **0.002** |
| 6-12 years (91) | 61 (67.0) | 30 (33.0) | **2.49 (1.16, 5.32)** | **0.019** | 2.21 (0.88, 5.54) | 0.093 |
| More than 12 years (40) | 18 (45.0) | 22 (55.0) | Ref | Ref | Ref | Ref |
| **Size of the household/ Family size** | | | | | | |
| 1 to 4 members (Small) (106) | 64 (60.4) | 42 (39.6) | Ref | Ref | Ref | Ref |
| 5 to 8 members (Medium) (135) | 101 (74.8) | 34 (25.2) | **1.95 (1.13, 3.38)** | **0.017** | **2.07 (1.02, 4.21)** | **0.045** |
| 9 and more members (Large) (21) | 15 (71.4) | 6 (28.6) | 1.64 (0.59, 4.57) | 0.343 | 1.54 (0.41, 5.83) | 0.522 |
| **Economically active members in the household (members with income source)** | | | | | | |
| None (8) | 6 (75.0) | 2 (25.0) | 1.90 (0.35, 10.17) | 0.456 |  |  |
| One (96) | 72 (75.0) | 24 (25.0) | 1.90 (0.95, 3.77) | 0.069 |  |  |
| Two (96) | 64 (66.7) | 32 (33.3) | 1.26 (0.65, 2.45) | 0.491 |  |  |
| Three or more (62) | 38 (61.3) | 24 (38.7) | Ref | Ref |  |  |
| **Household monthly income (NRs.)**Mean (SD) | | | | | | |
| Up to 50000 (159) | 119 (74.8) | 40 (25.2) | **2.05 (1.20, 3.49)** | **0.008** | **2.29 (1.22, 4.31)** | **0.010** |
| Above 50000 (103) | 61 (59.2) | 42 (40.8) | Ref | Ref | Ref | Ref |
| **Cancer diagnosed** | | | | | | |
| Breast Cancer (59) | 39 (66.1) | 20 (33.9) | 1.00 (0.51, 1.96) | 0.995 |  |  |
| Cervical Cancer (38) | 26 (68.4) | 12 (31.6) | 1.11 (0.51, 2.46) | 0.790 |  |  |
| Lung Cancer (42) | 32 (76.2) | 10 (23.8) | 1.64 (0.73, 3.71) | 0.231 |  |  |
| Prostate Cancer (14) | 11 (78.6) | 3 (21.4) | 1.88 (0.50, 7.17) | 0.353 |  |  |
| Others* (109) | 72 (66.1) | 37 (33.9) | Ref | Ref |  |  |
| **Duration since cancer diagnosis** | | | | | | |
| More than 2 years (25) | 17 (68.0) | 8 (32.0) | 0.94 (0.37, 22.38) | 0.888 |  |  |
| 1-2 years (52) | 39 (75.0) | 13 (25.0) | 1.32 (0.62, 2.79) | 0.468 |  |  |
| 6-12 months (77) | 49 (63.6) | 28 (36.4) | 0.77 (0.42, 1.43) | 0.408 |  |  |
| Less than 6 months (108) | 75 (69.4) | 33 (30.6) | Ref | Ref |  |  |
| **Stage of cancer at the time of diagnosis** | | | | | | |
| First (59) | 26 (44.1) | 33 (55.9) | Ref | Ref | Ref | Ref |
| Second (94) | 67 (71.3) | 27 (28.7) | **3.15 (1.59, 6.22)** | **<0.001** | 4.38 (1.98, 9.69) | **<0.001** |
| Third (85) | 67 (78.8) | 18 (21.2) | **4.72 (2.27, 9.82)** | **<0.001** | 4.45 (1.98, 10.01) | **<0.001** |
| Fourth (24) | 20 (83.3) | 4 (16.7) | **6.35 (1.93, 20.87)** | **0.002** | 7.53 (1.84, 30.87) | **0.005** |
| **Presence of any other chronic disease** | | | | | | |
| No (216) | 149 (69.0) | 67 (31.0) | Ref | Ref |  |  |
| Yes (46) | 31 (67.4) | 15 (32.6) | 0.93 (0.47, 1.84) | 0.833 |  |  |
| **Insurance under NHIP** | | | | | | |
| Insured (101) | 68 (67.3) | 33 (32.7) | Ref | Ref |  |  |
| Not insured (161) | 112 (69.6) | 49 (30.4) | 1.11 (0.65, 1.89) | 0.704 |  |  |
| **Received subsidies (Bipanna Nagarik Kosh and/or Provincial subsidies)** | | | | | | |
| No (21) | 18 (85.7) | 3 (14.3) | 2.93 (0.84, 10.23) | 0.093 |  |  |
| Yes (241) | 162 (67.2) | 79 (32.8) | Ref | Ref |  |  |
| **Type of health facility visited** | | | | | | |
| Public (143) | 104 (72.7) | 39 (27.3) | 0.66 (0.39, 1.12) | 0.124 |  |  |
| Private (119) | 76 (63.9) | 43 (36.1) | Ref | Ref |  |  |
| **Number of health facilities visited for cancer management** | | | | | | |
| One (70) | 48 (68.6) | 22 (31.4) | Ref | Ref |  |  |
| Two (85) | 51 (60.0) | 34 (40.0) | 0.69 (0.35, 1.34) | 0.270 |  |  |
| Three (60) | 48 (80.0) | 12 (20.0) | 1.83 (0.82, 4.12) | 0.142 |  |  |
| Four or more (47) | 33 (70.2) | 14 (29.8) | 1.08 (0.48, 2.41) | 0.850 |  |  |
| **OOPE** | | | | | | |
| No (110) | 77 (70.0) | 33 (30.0) | Ref | Ref |  |  |
| Yes (152) | 103 (67.8) | 49 (32.2) | 0.90 (0.53, 1.53) | 0.700 |  |  |
| **CATA (n=232)** | | | | | | |
| No (122) | 84 (68.9) | 38 (31.1) | Ref | Ref |  |  |
| Yes (110) | 76 (69.1) | 34 (30.9) | 1.01 (0.58, 1.77) | 0.969 |  |  |
| **Impoverishment (n= 177)** | | | | | | |
| No (106) | 74 (69.8) | 32 (30.2) | Ref | Ref |  |  |
| Yes (71) | 45 (63.4) | 26 (36.6) | 0.75 (0.40, 1.41) | 0.372 |  |  |
