# Supplementary material for: Psychological distress and its associated factors among cancer patients in Nepal: A cross-sectional study
Source: PLOS Ment Health. 2026 Mar 6;3(3):e0000419. doi: 10.1371/journal.pmen.0000419 (PMC12965590; doi:10.1371/journal.pmen.0000419)
Supplement: S4 Table — (DOCX) [file pmen.0000419.s009.docx]

**S4 Table. Bivariate and multivariate analysis for socio-demographic and clinical characteristic variables with stress symptoms**

| **Patients’ characteristics** | **Stress symptoms** | | **Model I,** | **p-value** | **Model II,** | **p-value** |
| --- | --- | --- | --- | --- | --- | --- |
|  | **Yes** | **No** | **cOR (95%CI)** |  | **aOR (95%CI)** |  |
|  | **n (%)** | **n (%)** |  |  |  |  |
| **Age (in years)** Mean (SD): 53.61 (14.41) | | | | | | |
| Less than 40 (50) | 23 (46.0) | 27 (54.0) | Ref | Ref | Ref | Ref |
| 40-59 (126) | 71 (56.3) | 55 (43.7) | 1.52 (0.79, 2.93) | 0.216 | 0.90 (0.39, 2.10) | 0.807 |
| 60 and above (86) | 58 (67.4) | 28 (32.6) | **2.43 (1.19, 4.98)** | **0.015** | 1.14 (0.44, 2.94) | 0.790 |
| **Sex** | | | | | | |
| Male (97) | 55 (56.7) | 42 (43.3) | Ref | Ref |  |  |
| Female (165) | 97 (58.8) | 68 (41.2) | 1.09 (0.66, 1.81) | 0.741 |  |  |
| **Marital status** | | | | | | |
| Single (25) | 9 (36.0) | 16 (64.0) | Ref | Ref | Ref | Ref |
| Married (218) | 130 (59.6) | 88 (40.4) | **2.63 (1.11, 6.21)** | **0.028** | 2.75 (0.86, 8.82) | 0.089 |
| Widowed (19) | 13 (68.4) | 6 (31.6) | **3.85 (1.09, 13.66)** | **0.037** | 2.25 (0.46, 10.99) | 0.316 |
| **Occupation status after cancer diagnosis** | | | | | | |
| Employed (61) | 28 (45.9) | 33 (54.1) | Ref | Ref | Ref | Ref |
| Unemployed (201) | 124 (61.7) | 77 (38.3) | **1.90 (1.06, 3.38)** | **0.030** | 1.65 (0.87, 3.14) | 0.129 |
| **Level of education** | | | | | | |
| Up to 5 years (131) | 85 (64.9) | 46 (35.1) | **2.77 (1.34, 5.74)** | **0.006** | 1.57 (0.62, 3.96) | 0.341 |
| 6-12 years (91) | 51 (56.0) | 40 (44.0) | 1.91 (0.90, 4.07) | 0.093 | 1.17 (0.47, 2.92) | 0.740 |
| More than 12 years (40) | 16 (40.0) | 24 (60.0) | Ref | Ref | Ref | Ref |
| **Size of the household/ Family size** | | | | | | |
| 1 to 4 members (Small) (106) | 53 (50.0) | 53 (50.0) | Ref | Ref | Ref | Ref |
| 5 to 8 members (Medium) (135) | 87 (64.4) | 48 (35.6) | **1.81 (1.08, 3.05)** | **0.025** | 1.53 (0.83, 2.80) | 0.171 |
| 9 and more members (Large) (21) | 12 (57.1) | 9 (42.9) | 1.33 (0.52, 3.43) | 0.550 | 0.90 (0.30, 2.73) | 0.854 |
| **Economically active members in the household (members with income source)** | | | | | | |
| None (8) | 2 (25.0) | 6 (75.0) | 0.28 (0.05, 1.47) | 0.131 |  |  |
| One (96) | 59 (61.5) | 37 (38.5) | 1.31 (0.69, 2.51) | 0.409 |  |  |
| Two (96) | 57 (59.4) | 39 (40.6) | 1.20 (0.63, 2.29) | 0.573 |  |  |
| Three or more (62) | 34 (54.8) | 28 (45.2) | Ref | Ref |  |  |
| **Household monthly income (NRs.)**Mean (SD) | | | | | | |
| Up to 50000 (159) | 98 (61.6) | 61 (38.4) | 1.46 (0.88, 2.41) | 1.141 |  |  |
| Above 50000 (103) | 54 (52.4) | 49 (47.6) | Ref | Ref |  |  |
| **Cancer diagnosed** | | | | | | |
| Breast Cancer (59) | 38 (64.4) | 21 (35.6) | 1.65 (0.86, 3.17) | 0.132 |  |  |
| Cervical Cancer (38) | 20 (52.6) | 18 (47.4) | 1.01 (0.48, 2.12) | 0.971 |  |  |
| Lung Cancer (42) | 27 (64.3) | 15 (35.7) | 1.64 (0.79, 3.42) | 0.186 |  |  |
| Prostate Cancer (14) | 10 (71.4) | 4 (28.6) | 2.28 (0.67, 7.18) | 0.185 |  |  |
| Others* (109) | 57 (52.3) | 52 (47.7) | Ref | Ref |  |  |
| **Duration since cancer diagnosis** | | | | | | |
| More than 2 years (25) | 15 (60.0) | 10 (40.0) | 1.11 (0.46, 2.70) | 0.813 |  |  |
| 1-2 years (52) | 36 (69.2) | 16 (30.8) | 1.67 (0.83, 3.37) | 0.152 |  |  |
| 6-12 months (77) | 39 (50.6) | 38 (49.4) | 0.76 (0.42, 1.37) | 0.363 |  |  |
| Less than 6 months (108) | 62 (57.4) | 46 (42.6) | Ref | Ref |  |  |
| **Stage of cancer at the time of diagnosis** | | | | | | |
| First (59) | 28 (47.5) | 31 (52.5) | Ref | Ref | Ref | Ref |
| Second (94) | 54 (57.4) | 40 (42.6) | 1.50 (0.78, 2.88) | 0.229 | 1.50 (0.74, 3,06) | 0.262 |
| Third (85) | 52 (61.2) | 33 (38.8) | 1.75 (0.89, 3.42) | 0.104 | 1.55 (0.74, 3.24) | 0.247 |
| Fourth (24) | 18 (75.0) | 6 (25.0) | **3.32 (1.16, 9.55)** | **0.026** | **3.47 (1.03, 11.66)** | **0.044** |
| **Presence of any other chronic disease** | | | | | | |
| No (216) | 126 (58.3) | 90 (41.7) | Ref | Ref |  |  |
| Yes (46) | 26 (56.5) | 20 (43.5) | 0.93 (0.49, 1.77) | 0.821 |  |  |
| **Insurance under NHIP** | | | | | | |
| Insured (101) | 53 (52.5) | 48 (47.5) | Ref | Ref |  |  |
| Not insured (161) | 99 (61.5) | 62 (38.5) | 1.45 (0.87, 2.39) | 0.151 |  |  |
| **Received subsidies (Bipanna Nagarik Kosh and/or Provincial subsidies)** | | | | | | |
| No (21) | 15 (71.4) | 6 (28.6) | 1.90 (0.71, 5.06) | 0.200 |  |  |
| Yes (241) | 137 (56.8) | 104 (43.2) | Ref | Ref |  |  |
| **Type of health facility visited** | | | | | | |
| Public (143) | 76 (53.1) | 67 (46.9) | 1.56 (0.95, 2.56) | 0.081 |  |  |
| Private (119) | 76 (63.9) | 43 (36.1) | Ref | Ref |  |  |
| **Number of health facilities visited for cancer management** | | | | | | |
| One (70) | 42 (60.0) | 28 (40.0) | Ref | Ref |  |  |
| Two (85) | 45 (52.9) | 40 (47.1) | 0.75 (0.40, 1.42) | 0.379 |  |  |
| Three (60) | 42 (70.0) | 18 (30.0) | 1.56 (0.75, 3.23) | 0.236 |  |  |
| Four or more (47) | 23 (48.9) | 24 (51.1) | 0.64 (0.30, 1.35) | 0.239 |  |  |
| **OOPE** | | | | | | |
| No (110) | 62 (56.4) | 48 (43.6) | Ref | Ref |  |  |
| Yes (152) | 90 (59.2) | 62 (40.8) | 1.12 (0.68, 1.85) | 0.645 |  |  |
| **CATA (n=232)** | | | | | | |
| No (122) | 70 (57.4) | 52 (42.6) | Ref | Ref |  |  |
| Yes (110) | 63 (57.3) | 47 (42.7) | 0.99 (0.59, 1.68) | 0.987 |  |  |
| **Impoverishment (n= 177)** | | | | | | |
| No (106) | 58 (54.7) | 48 (45.3) | Ref | Ref |  |  |
| Yes (71) | 46 (64.8) | 25 (35.2) | 1.52 (0.82, 2.83) | 0.183 |  |  |
