# Supplementary material for: Psychological distress and its associated factors among cancer patients in Nepal: A cross-sectional study
Source: PLOS Ment Health. 2026 Mar 6;3(3):e0000419. doi: 10.1371/journal.pmen.0000419 (PMC12965590; doi:10.1371/journal.pmen.0000419)
Supplement: Attachment — Submitted filename: Response to Reviewers.docx [file pmen.0000419.s011.docx]

**07/09/2025**

**Dear Editor and Reviewers,**

We sincerely thank you and the reviewers for the time and effort spent evaluating our manuscript. We greatly appreciate the constructive comments and suggestions, which have helped us improve the clarity, rigor, and overall quality of our work. Below, we provide a point-by-point response to each comment. All changes have been incorporated into the revised manuscript, and relevant line numbers in the updated version are indicated. In addition, too the comments and suggestions received, minor grammatical and other necessary revisions have been made under track change.

Thank you!

**Kind regards,**

**Ankit Acharya**

**On behalf of all co-authors.**

**Response to editor’s comments**

1. Please ensure that your Ethics Statement is available in its entirety at the beginning of your Methods section, under a subheading 'Ethics Statement'.

**Response- We have now included the ethics statement at the beginning of the methods section, under a subheading ‘Ethics Statement’.**

2. We have noticed that you have uploaded Supporting Information files, but you have not included a list of legends. Please add a full list of legends for your Supporting Information files after the references list.

**Response- We have now ensured that the uploaded Supporting Information files are now included with a list of legends after the references.**

3. We note that there is identifying data in the Supporting Information file “Dataset.xlsx”. Due to the inclusion of these potentially identifying data, we have removed this file from your file inventory. Prior to sharing human research participant data, authors should consult with an ethics committee to ensure data are shared in accordance with participant consent and all applicable local laws.

**Response- We have revised our submission accordingly. The file “Dataset.xlsx” containing potentially identifying data has been removed, and we acknowledge that any future data sharing will be undertaken only after consultation with the ethics committee to ensure compliance with participant consent and local regulations. An anonymized dataset has now been provided as “S1 Dataset_Anonymized.xlsx.”**

**Response to first reviewer’s comments**

Reviewer #1: 1. The article provides a clear and data-driven rationale for the study, establishing the growing burden of cancer globally and in Nepal. It effectively contextualizes both the clinical and psychosocial dimensions of cancer, with a specific focus on the intersection of financial hardship and psychological distress which is well articulated and highly relevant, especially for low- and middle-income country contexts where cancer care infrastructure is often limited.

**Response- Thank you for your thorough comments. We agree to these critical reflections.**

2. While the inclusion of global and national statistics is important, the opening paragraphs could benefit from slight condensation to avoid overwhelming the reader with numbers. Consider prioritizing the most critical figures and integrating them more smoothly into the narrative.

**Response- The opening section has been condensed by reducing the number of global statistics presented, prioritizing only the most critical figures to improve clarity and narrative flow.**

3. The relationship between the "six D’s" and psychological distress could be explained more explicitly. Currently, the transition from physical to psychosocial consequences feels abrupt.

**Response- We have clarified the connection between the "six D’s" and psychological distress in lines 65-70.**

4. Abbreviations like OOPE and CATA are introduced without prior definition. These should be spelled out when first mentioned to ensure clarity for readers unfamiliar with the terms.

**Response- Abbreviations have now been defined when first mentioned in lines 72–77, to ensure clarity for all readers.**

5. The challenges in the Nepalese context are noted, but the rationale for focusing on tertiary-level referral hospitals could be further strengthened. Briefly discuss their significance in the healthcare system and why they are a suitable setting for this study.

**Response- The manuscript has been revised (lines 109–113) to clarify the rationale for selecting tertiary-level referral hospitals, highlighting their central role in the Nepalese healthcare system and their suitability as sites for capturing a comprehensive spectrum of cancer care.**

6. Ensure that all claims—especially those linking treatment side effects to psychological distress—are supported by high-quality, recent references. This will bolster the credibility of the argument.

**Response- Updated in lines 78-82.**

7. The rationale for analyzing 262 participants (out of 400) is well explained, but consider discussing potential bias introduced by excluding non-DASS-21 respondents (e.g., were there notable differences?). Sampling procedure should be described more clearly. It is not clear how patients were selected from the hospital population—was it random, purposive, or convenience sampling?

**Response- Limitations regarding potential bias from excluding non-DASS-21 respondents have been addressed in lines 399-404. Additionally, the sampling procedures have been clarified in lines 140-148, specifying the method used to select patients from the hospital population.**

8. The sample size formula is included but incomplete. Either present the full derivation with parameter values or simplify and state that it was calculated based on expected effect size and power. P-value should be corrected: “P-values less than 0.05 was considered as significant” should read “were considered significant.”

**Response- The manuscript has been revised to include the complete sample size formula with full derivation and parameter values (lines 120–139 and 149–152). The P-value statement has been corrected in line 174 to read: “Statistical significance was defined as a two-sided p < 0.05.”**

9. The DASS-21 tool is well-described, but more information on its validation in the Nepali context would enhance credibility (e.g., reliability coefficients, cultural appropriateness). Clarify if interviews were conducted by trained enumerators and whether there was any quality control (e.g., supervisor checks, data audits).

**Response- Information on the validation of DASS-21 in the Nepali context has been added (lines 157–160). Additionally, clarification regarding interviews being conducted by trained enumerators, along with quality control measures such as supervisor checks and data audits, has been provided in lines 162–166.**

10. The operationalization of psychological distress and financial indicators is clear and appropriate. However, grouping categories for logistic regression should be justified—for example, why >9 on depression is chosen as a cutoff.

**Response- Justification for the cut-off scores used for depression, anxiety, and stress has been provided in lines 190-194.**

11. The ethical procedures are appropriately described. However, mention how participant privacy was protected during face-to-face interviews in hospital settings (e.g., private rooms?).

**Response- Details regarding participants’ privacy protection has been shared in the lines 162-166.**

12. Consider linking demographics to potential risk factors for psychological distress in the discussion section. Clarify the distribution of participants across the two hospitals.

**Response- Demographics have now been linked to potential risk factors for psychological distress in the discussion section (lines 339-363). The distribution of participants across the two hospitals has also been clarified (lines 205-206).**

13. Clarify figures for cancer stages in lines 185–186: Include the percentage for each stage (e.g., stage 1, 2, 3, 4) for completeness. Line 189: Rephrase to clarify—e.g., "The mean number of chemotherapy and radiotherapy cycles completed was 6.63 (SD = 5.01) and 15.71 (SD = 11.41), respectively." The data on multiple health facility use (lines 198–200) should be expanded with potential interpretation—what might this imply (e.g., care-seeking behavior, referrals, fragmented care)?

**Response- Figures has been clarified for cancer stages in the lines 226-228 with percentage included; rephased in the lines 228-233; data on multiple health facility use has been expanded with interpretation in the lines 379-384.**

14. Add comparative reference to national or global averages for psychological distress among cancer patients to contextualize these findings. Briefly describe what the DASS-21 thresholds mean for each category (especially in figure captions or supplemental materials, lines 206-208).

**Response- Comparative reference with global averages for psychological distress has been added to contextualize the findings in the lines 327-333. DASS-21 thresholds have been described in the figure captions.**

15. Improve narrative clarity: Instead of repeating “In reference to...”, consider a tabular summary with narrative highlights for readability. Model II is mentioned multiple times, but results are only partially elaborated. Explicitly state which variables remained significant and how the strength of association changed after adjustment. It would be helpful to briefly define each model before presenting the results (e.g., "Model I includes unadjusted estimates, Model II includes adjusted variables", line 212 and so on).

**Response- We greatly appreciate this comment. Revision has been made in the results sections accordingly. Each model has now been explicitly defined prior to presenting results, and the description of Model II has been expanded to clearly state which variables remained significant and how the strength of association changed after adjustment.**

16. Some sections are repetitive or overly descriptive (e.g., lines 264–275). Condense citations or summarize patterns with fewer studies. The explanation about family size not being significant (lines 293–295) is speculative—this should be clearly marked as a hypothesis, or replaced with a suggestion for future study. The claim that financial burden is not associated with distress (lines 313–319) is surprising and contrasts with global literature. This result needs: a more thorough explanation (e.g., potential limitations in measurement) and a discussion on possible underreporting or cultural resilience factors.

**Response- We thank the reviewer for these thoughtful comments. Repetitive and overly descriptive sections have been condensed to improve clarity and flow (lines 322-335). The explanation regarding family size has been revised to highlight it as a suggestion for future study (lines 355-359, 360-363). Furthermore, the unexpected finding that financial burden was not associated with distress has been discussed in greater detail, including potential limitations in measurement, underreporting, and cultural resilience factors, with recommendations for future research (lines 385–398).**

17. Reframe “psychosocial costs should not be one of the costs patients pay” (line 331). It’s powerful but could be more specific—e.g., "Psychological distress must be recognized and mitigated as part of holistic cancer care." Expand slightly on how the cancer control strategy (2024–2030) can support mental health—e.g., training counselors, routine distress screening, or including mental health in financial protection packages.

**Response- The statement has been reframed for greater specificity (lines 411-413), and further elaboration has been added on how the Nepal Cancer Control Strategy (2024-2030) can address mental health needs, including training of counselors, routine distress screening, and integration of mental health into financial protection measures (lines 413-426).**

18. Improve the layout of the tables.

**Response- The layout of all tables has been revised for improved clarity, readability, and presentation.**

**Response to second reviewer’s comments**

Although the manuscript is presented in an intelligible fasion and written in standard English, the manuscript is missing pertinent details such as if a particular sampling method will be used to recruit participants, if an inclusion and exclusion criteria will be implored, and how the sample size was determined.

**Response- Thank you for your comments. We have now revised the manuscript with details of sampling method used to recruit participants, inclusion and exclusion criteria used, and steps of sample size determination.**
