# Supplementary material for: Psychological distress and its associated factors among cancer patients in Nepal: A cross-sectional study
Source: PLOS Ment Health. 2026 Mar 6;3(3):e0000419. doi: 10.1371/journal.pmen.0000419 (PMC12965590; doi:10.1371/journal.pmen.0000419)
Supplement: Attachment — Submitted filename: Response_to_Reviewers_auresp_2.docx [file pmen.0000419.s012.docx]

**27/10/2025**

**Dear Editor and Reviewers,**

We sincerely thank you all for the time and effort spent in evaluating our manuscript. We greatly appreciate the constructive comments and suggestions, which have helped us improve the clarity, rigor, and overall quality of our work.

Below, we provide a point-by-point response to each comment. All changes have been incorporated into the revised manuscript, and relevant line numbers in the updated version are indicated.

Thank you for your insightful and valued contributions!

**Kind regards,**

**Ankit Acharya**

**On behalf of all co-authors.**

**Response to editor’s comments**

**Reviewer #1:** The manuscript addresses an important and under-researched area- psychological distress among cancer patients in Nepal. The study is relevant and timely, especially in low-resource settings. However, the manuscript requires:
**Response: Kindly accept our sincere thanks for highlighting the importance of our study on psychological distress among cancer patients in Nepal. We appreciate the encouraging comments on the study’s relevance and timeliness.**

1. Language polishing, improved coherence, and clarifications in methods and results to enhance its readability and scientific rigor.
**Response: Thank you for your constructive feedback highlighting the need for linguistic refinement and improved coherence and clarity. The entire manuscript has been carefully edited for language accuracy and coherence across sections has been strengthened. Explanations in the Methods and Results sections have been clarified to strengthen scientific rigor. These changes have improved the readability and presentation of the paper.**

2. More citations or statistics to support the claim that psychological distress is frequently overlooked.
**Response: Additional citations and supporting statistics have been incorporated (lines 77-85) to support the claim that psychological distress among cancer patients is often under-recognized, particularly in low-resource contexts.**

Despite substantial global evidence highlighting the unmet psychosocial needs of individuals with cancer (19-21), 40-90% of patients with psychological distress remain unrecognized by clinicians, leading to inadequate care (22-24). A recent systematic review of cancer populations in Southeast Asia found that the prevalence of anxiety ranged from 7 to 88 percent and depression from 3 to 65 percent, with most studies documenting moderate to severe distress but limited screening and counselling integration in clinical practice (7). Moreover, while global research increasingly conceptualizes “financial toxicity” as a determinant of psychological well‑being (12, 25), no study in Nepal has systematically analyzed how financial protection indicators such as OOPE, CATA, and impoverishment relate to psychological distress among cancer patients.

3. Clarity in the gaps that this study fills.
**Response: The research gap is now explicitly framed in relation to Nepal, supported by newly added global and regional literatures (lines 77-85), clarifying both the empirical and contextual novelty of the present study (lines 86-93).**

**Lines 77-85**

Despite substantial global evidence highlighting the unmet psychosocial needs of individuals with cancer (19-21), 40-90% of patients with psychological distress remain unrecognized by clinicians, leading to inadequate care (22-24). A recent systematic review of cancer populations in Southeast Asia found that the prevalence of anxiety ranged from 7 to 88 percent and depression from 3 to 65 percent, with most studies documenting moderate to severe distress but limited screening and counselling integration in clinical practice (7). Moreover, while global research increasingly conceptualizes “financial toxicity” as a determinant of psychological well‑being (12, 25), no study in Nepal has systematically analyzed how financial protection indicators such as OOPE, CATA, and impoverishment relate to psychological distress among cancer patients.

**Lines 86-93**

Existing literature in Nepal has either focused primarily on estimating financial burden across NCDs, including cancer (26-28), or has explored psychological distress without integrating indicators of financial protection (29-32). This study therefore fills an important gap by providing the first evidence linking financial hardship and psychological well-being in the context of cancer care in Nepal. By integrating psycho-social and economic perspectives within a low-resource health system context, this study contributes new evidence to the growing international effort to embed mental-health screening and financial protection within comprehensive, person-centered cancer care frameworks.

4. Caution with interpretation, particularly with statistical association, causation should not be implied. Also, consider expanding on why no association was found- could it be due to measurement issues or coping mechanisms?
**Response: We have revised the Results (Lines 239-240); and Discussion (lines 328-330; 335-338; 401-407; 411-429) to ensure cautious interpretation of statistical findings and to avoid causal implications. Further elaboration has been provided on potential reasons for the absence of significant associations, including contextual coping strategies and methodological considerations related to measurement precision.**

**Lines 239-240**

Higher levels of depression, anxiety, and stress were associated with more advanced stages of cancer.

**Lines 328-330**

In this study, participants aged 60 years and older had significantly higher odds of reporting depression, anxiety, and stress than younger individuals, demonstrating a strong association between older age and psychological distress.

**Lines 335-338**

Lack of a stable income may heighten financial insecurity and psychological dependence, which in turn are associated with reduced self‑esteem and greater emotional distress, consistent with evidence that persistent financial hardship can diminish self‑esteem and contribute to depressive symptoms (51).

**Lines 401-407**

Alternatively, following a cancer diagnosis, immediate psychological impact of treatment‑related expenses may be counterbalanced by structural and social buffering mechanisms such as government subsidies, health insurance support, and informal family financing. Moreover, enduring cultural values in Nepal that prioritize resilience, collective responsibility, and acceptance may attenuate the perceived connection between economic hardship and emotional distress, thereby reducing the observable statistical association between financial burden and psychological outcomes.

**Lines 411-429**

This study has several limitations that should be considered when interpreting the findings. First, the relatively small analytic sample may have reduced statistical power to detect associations and limited the generalizability of results beyond the study sites. The cross‑sectional design restricts causal inference between psychological outcomes and explanatory variables. The use of face‑to‑face interviews may have introduced social desirability bias, as some participants might have under‑reported or modified their emotional experiences to align with perceived expectations of the interviewer. Non‑response and self‑selection bias is also possible, as individuals experiencing greater illness severity or psychological distress may have declined participation owing to fatigue, stigma, or discomfort. Consequently, the reported prevalence of distress may either underestimate or overestimate the true population burden. Second, psychological distress was measured using a self‑reported screening tool rather than clinical evaluation, which may have introduced measurement and cultural response biases. Moreover, the insignificant associations observed between financial hardship indicators (OOPE, CATA, and impoverishment) and psychological distress may partly reflect methodological limitations. Financial hardship is a multidimensional construct that involves material, psychological, and behavioral responses to economic strain, and inconsistencies in its measurement can substantially influence observed relationships (69). In this study, financial data were collected at a single cross‑sectional time point, which may not have coincided with peak treatment phases or periods of acute economic pressure, thereby underestimating the short‑term psychological impact of financial stress (69, 70).

5. Recommendations are important but should be more concise and clearly actionable. **Response: We acknowledge the reviewer’s recommendation and have revised the concluding section (lines 436- 447) to present the implications in a more concise, focused, and actionable manner. The recommendations now emphasize practical strategies relevant to clinicians, policymakers, and future researchers in low-resource oncology settings.**

In light of our findings, routine mental-health screening can be incorporated into institutional cancer-care pathways to facilitate early identification and management of psychological distress. Oncology nurses and allied health personnel can be trained in basic psychosocial counselling and communication skills to provide immediate support and facilitate appropriate referrals for both patients and their caregivers, recognizing their shared psychological burden throughout the course of illness.

These actions align with the Nepal Cancer Control Strategy (2024- 2030), which emphasizes enhanced capacity in diagnosis, treatment, and rehabilitation, and the National Health Financing Strategy (2023- 2033), which aims to reduce financial and social vulnerability caused by illness (71, 72). Integrating psychosocial screening and counselling within these national frameworks would help mitigate both the psychological and economic burden of cancer and advance equitable, person‑centered oncology care in Nepal.

**Reviewer #3:** The abstract is wordy and has some awkward phrasing (“Patients at no cost should undergo through psychological distress” is unclear). It needs language refinement

**We appreciate the reviewer’s observation regarding clarity and concision. The abstract has been carefully rewritten to improve readability, remove ambiguous phrasing, and enhance overall coherence. Particular attention has been paid to refining awkward expressions to convey the study’s intent with precision and fluency. See lines 20-49.**

**Background**

Psychological distress, encompassing depression, anxiety, and stress, is common among individuals with cancer but remains inadequately recognized in many low- and middle-income countries, including Nepal. Despite growing emphasis on holistic cancer care, the psychological dimensions of cancer management are often overshadowed by clinical and financial concerns. This study aimed to estimate the prevalence of psychological distress among cancer patients in Nepal and to identify its associated sociodemographic, clinical, and economic factors.

**Methodology**

This study was conducted among cancer patients in two largest tertiary level referral hospitals in Nepal. The validated Nepali version of the Depression, Anxiety and Stress Scale (DASS‑21) was used to assess psychological distress. A total of 262 participants were enrolled in this study. SPSS version 27 was used to perform cleaning, coding and analyses. P-values of less than 0.05 indicated significant associations. Odds ratios were calculated using a binary logistic regression model, and necessary adjustments was made in the multivariate logistic regression model.
**Results**
More than two-thirds of cancer patients had depression (66.8%) and anxiety (68.7%) symptoms, and nearly three-fifths had symptoms of stress (58.0%). Higher odds of psychological distress were observed among participants who were older, unemployed, had fewer years of education, and had advanced-stage cancer. In reference to those who were diagnosed with first stage of cancer, those who were diagnosed with second, third and fourth stage of cancer had 2.75 higher odds (cOR 2.75; 95% CI 1.39, 5.42; p-value = 0.004), 2.81 higher odds (cOR 2.81; 95% CI 1.40, 5.64; p-value = 0.004) and 4.21 higher odds (cOR 4.21; 95% CI 1.39, 12.76; p-value = 0.011) of having depressive symptoms respectively. Despite that households of cancer patients bear out of pocket expense, catastrophic health expense and impoverishment, no significant association was found among these indicators and psychological distress.
**Conclusion**
Psychological distress is highly prevalent among Nepali cancer patients and is linked primarily to sociodemographic and clinical factors rather than direct economic burden. Integrating routine mental health screening and basic counseling into oncology services is urgently needed to improve patient’s overall well-being.

The introduction is comprehensive but overburdened with detail (especially about radiotherapy and chemotherapy side effects). It risks losing focus on the rationale for the study. It must be streamlined.
The stated research gap is still vague. You note that distress is “least understood and often ignored,” but don’t explicitly show how prior Nepal-specific or South Asian evidence is lacking. More local or regional citations are needed.
**Response: Redundant descriptions of radiotherapy and chemotherapy side effects have been reduced. Refer to the lines 75-76.**

It is also influenced by side effects of treatment measures such as Radiotherapy (13-15), and Chemotherapy (7, 16-18).

**The research gap is now explicitly framed in relation to Nepal, supported by newly added global and regional literatures (lines 77-85), clarifying both the empirical and contextual novelty of the present study (lines 86-93).**

**Lines 77-85**

Despite substantial global evidence highlighting the unmet psychosocial needs of individuals with cancer (19-21), 40-90% of patients with psychological distress remain unrecognized by clinicians, leading to inadequate care (22-24). A recent systematic review of cancer populations in Southeast Asia found that the prevalence of anxiety ranged from 7 to 88 percent and depression from 3 to 65 percent, with most studies documenting moderate to severe distress but limited screening and counselling integration in clinical practice (7). Moreover, while global research increasingly conceptualizes “financial toxicity” as a determinant of psychological well‑being (12, 25), no study in Nepal has systematically analyzed how financial protection indicators such as OOPE, CATA, and impoverishment relate to psychological distress among cancer patients.

**Lines 86-93**

Existing literature in Nepal has either focused primarily on estimating financial burden across NCDs, including cancer (26-28), or has explored psychological distress without integrating indicators of financial protection (29-32). This study therefore fills an important gap by providing the first evidence linking financial hardship and psychological well-being in the context of cancer care in Nepal. By integrating psycho-social and economic perspectives within a low-resource health system context, this study contributes new evidence to the growing international effort to embed mental-health screening and financial protection within comprehensive, person-centered cancer care frameworks.

The sampling description is complex. While two-stage random sampling is commendable, it’s not clear how representative the final sample of 262 is, given that 400 were originally recruited. Potential selection bias from those who declined DASS-21 should be acknowledged more strongly.
The sample size calculation is overly technical for the main text and doesn’t clearly link to the actual analytic sample. Why was a “two independent groups” formula used when this is a descriptive cross-sectional study? Simplification and better justification would help.
Training of enumerators (four hours on Zoom) seems minimal for sensitive psychological assessment. This limitation should be acknowledged.
Operational definitions are useful, but repeating cutoffs for DASS-21 in such detail is redundant. Please move to supplementary material.
**Response: The potential for selection bias arising from participants who declined completion of the DASS-21 has now been explicitly discussed in the Methods section (lines 139-144) and subsequently revisited in the Limitations (lines 411-429).**

**Lines 139-144**

For this specific analysis focused on psychological distress, no stratification by facility type was performed. Of the 400 participants enrolled, 262 consented to and completed the Depression, Anxiety and Stress Scale - 21 Items (DASS-21) assessment tool for measuring psychological distress. The exclusion of non-respondents may have introduced non-response bias, which is acknowledged as a limitation. Analyses of economic outcomes from the broader study, using the same sample, will be reported separately (34).

**Lines 411-429**

This study has several limitations that should be considered when interpreting the findings. First, the relatively small analytic sample may have reduced statistical power to detect associations and limited the generalizability of results beyond the study sites. The cross‑sectional design restricts causal inference between psychological outcomes and explanatory variables. The use of face‑to‑face interviews may have introduced social desirability bias, as some participants might have under‑reported or modified their emotional experiences to align with perceived expectations of the interviewer. Non‑response and self‑selection bias is also possible, as individuals experiencing greater illness severity or psychological distress may have declined participation owing to fatigue, stigma, or discomfort. Consequently, the reported prevalence of distress may either underestimate or overestimate the true population burden. Second, psychological distress was measured using a self‑reported screening tool rather than clinical evaluation, which may have introduced measurement and cultural response biases. Moreover, the insignificant associations observed between financial hardship indicators (OOPE, CATA, and impoverishment) and psychological distress may partly reflect methodological limitations. Financial hardship is a multidimensional construct that involves material, psychological, and behavioral responses to economic strain, and inconsistencies in its measurement can substantially influence observed relationships (69). In this study, financial data were collected at a single cross‑sectional time point, which may not have coincided with peak treatment phases or periods of acute economic pressure, thereby underestimating the short‑term psychological impact of financial stress (69, 70).

**The sample size calculation has been simplified and rewritten for clarity (lines 125-138), with technical details relocated to the supplementary file (line number 129).**

**Lines 125-138**

This study has several limitations that should be considered when interpreting the findings. First, the relatively small analytic sample may have reduced statistical power to detect associations and limited the generalizability of results beyond the study sites. The cross‑sectional design restricts causal inference between psychological outcomes and explanatory variables. The use of face‑to‑face interviews may have introduced social desirability bias, as some participants might have under‑reported or modified their emotional experiences to align with perceived expectations of the interviewer. Non‑response and self‑selection bias is also possible, as individuals experiencing greater illness severity or psychological distress may have declined participation owing to fatigue, stigma, or discomfort. Consequently, the reported prevalence of distress may either underestimate or overestimate the true population burden. Second, psychological distress was measured using a self‑reported screening tool rather than clinical evaluation, which may have introduced measurement and cultural response biases. Moreover, the insignificant associations observed between financial hardship indicators (OOPE, CATA, and impoverishment) and psychological distress may partly reflect methodological limitations. Financial hardship is a multidimensional construct that involves material, psychological, and behavioral responses to economic strain, and inconsistencies in its measurement can substantially influence observed relationships (69). In this study, financial data were collected at a single cross‑sectional time point, which may not have coincided with peak treatment phases or periods of acute economic pressure, thereby underestimating the short‑term psychological impact of financial stress (69, 70).

**Line number 129**

The details of the sample size calculation are provided in the supplementary material (S2 Text).

**The description now directly links the analytic sample to study objectives, and the use of the “two independent groups” formula is succinctly justified in the lines 126-128.**

This sample size was calculated to detect small differences between subgroups (public and private hospitals) (effect size = 0.2) with 80% statistical power and a 5% level of significance (33).

**Further, we have acknowledged the brevity of enumerator training and its implications for data quality within the methodology (see lines 156-158).**

Although the training period was brief, the PI’s active involvement in the field, continuous supervision, and regular data audits likely mitigated any potential impacts on data consistency and ensured the completeness and accuracy of responses.

**Finally, to enhance readability, detailed operational definitions and DASS-21 cut‑off criteria have been moved from the main text to the supplementary material (lines 171-173).**

**DASS-21:** Refers to depression, anxiety, and stress scale, which consists of 21 items. Detailed scoring procedures and cut-off values for each subscale are provided as supplementary files (S3 Text).

The prevalence of distress is very high compared with global norms. This raises the question of whether cutoffs were too sensitive, or whether cultural/contextual factors inflated reporting. This issue is not discussed.
Tables are extremely dense and may overwhelm readers. Please move some to supplementary.
Reporting of missing data (e.g., why only 177 respondents for impoverishment) is buried. This needs to be clearer.
**Response: The discussion has been expanded to explore plausible reasons for the relatively high prevalence observed in this study, including contextual, cultural, and measurement‑related factors (lines 314-327).**

Although the Nepali version of the DASS‑21 has been psychometrically validated and shown to possess strong reliability and construct validity among Nepali adults (35), contextual factors may have influenced the reporting of symptoms. In South Asian contexts such as Nepal, the experience of cancer survival is shaped by limited psychosocial support and persistent uncertainty, where emotional distress is often expressed through physical symptoms such as pain, fatigue, and insomnia, leading to heightened attention to bodily sensations and greater overall psychological burden (48). Consistent with emerging cognitive‑affective models, such as the Cancer Threat Interpretation Model, pain and somatic signals may be cognitively appraised as potential signs of recurrence, reinforcing fear, worry, and maladaptive coping (49). Recent evidence also suggests that somatosensory amplification and fear of cancer recurrence mediate the relationship between bodily awareness and psychological distress (50). The high prevalence reported in this study may reflect a genuine psychosocial burden shaped by the interplay of cultural expression, stigma surrounding cancer and mental health, pain‑related threat appraisal, and scarcity of mental health integration within oncology services.

**Tables 4 and 5, which contained extensive detail, have been relocated to the supplementary material to improve narrative flow.**

**In addition, the reporting of missing data, including the explanation for 177 responses on impoverishment, has been clarified in the Methods section (lines 219-226).**

Unlike the analysis of CATA, which required data on total household consumption, food expenditure (to estimate subsistence spending), and OOPE, the assessment of impoverishment additionally required comprehensive pre‑ and post‑payment household expenditure information across all major categories defined by the Classification of Individual Consumption According to Purpose including food and non-food expenses, to identify movements across the poverty threshold. As this analysis necessitated complete and itemized consumption records, only 177 respondents provided sufficiently detailed data, resulting in a smaller analytic sample.

While you compare prevalence with global studies, the interpretation is somewhat superficial. Why is prevalence in Nepal higher? Structural issues (health system capacity, stigma, financial hardship, cultural perceptions of mental health) should be more deeply explored.
The null findings regarding OOPE, CATA, and impoverishment are glossed over. This is surprising given the strong theoretical link, and deserves a more thorough exploration (measurement error, timing of cost assessment, subsidies masking true burden, coping strategies, etc.).

**Response: The comparative discussion (lines 302-327) has been revised to engage more critically with global literature, elaborating on structural determinants such as limited mental‑health infrastructure, socioeconomic hardship, treatment‑related financial strain, and culturally embedded stigma.**

This study found a high prevalence of depression, anxiety, and stress among cancer patients, with more than two-thirds of cancer patients reporting depression and anxiety symptoms. Consistent to our findings, previous studies have reported that almost two-thirds of cancer patients had depression (38, 39). Comparable findings have been reported in other studies, where between one-third and two-thirds of cancer patients experienced depression and anxiety, though some studies noted prevalence rates as high as 90% (29-31, 38-45). An umbrella review of systematic reviews and meta-analyses reported a summary prevalence of depression among cancer survivors of 33.2% (95% CI: 27.6-38.7) and anxiety of 30.6% (95% CI: 24.0-37.1) (46). This indicates a comparatively greater psychological burden among Nepali cancer patients. With regard to stress, an integrated review reported that approximately 60-70% of cancer patients experience stress, which is broadly consistent with the present study, in which nearly three-fifths of patients reported stress (47). However, previous studies have shown wide variation, ranging from about one-fifth to nearly four-fifths (29, 31, 42, 44). Although the Nepali version of the DASS‑21 has been psychometrically validated and shown to possess strong reliability and construct validity among Nepali adults (35), contextual factors may have influenced the reporting of symptoms. In South Asian contexts such as Nepal, the experience of cancer survival is shaped by limited psychosocial support and persistent uncertainty, where emotional distress is often expressed through physical symptoms such as pain, fatigue, and insomnia, leading to heightened attention to bodily sensations and greater overall psychological burden (48). Consistent with emerging cognitive‑affective models, such as the Cancer Threat Interpretation Model, pain and somatic signals may be cognitively appraised as potential signs of recurrence, reinforcing fear, worry, and maladaptive coping (49). Recent evidence also suggests that somatosensory amplification and fear of cancer recurrence mediate the relationship between bodily awareness and psychological distress (50). The high prevalence reported in this study may reflect a genuine psychosocial burden shaped by the interplay of cultural expression, stigma surrounding cancer and mental health, pain‑related threat appraisal, and scarcity of mental health integration within oncology services.

**Furthermore, the section addressing null findings (lines 383-410) has been expanded to discuss potential explanations, including measurement timing, under‑reporting due to coping mechanisms, and possible masking of costs by partial subsidies.**

Much of the global literature has identified financial burden as a strong correlate of psychological distress (61-64). Contrary to our expectations, no significant association was observed between OOPE, CATA or impoverishment and psychological distress in this study. Several methodological and contextual factors may explain this finding. OOPE data are typically highly skewed, and the large standard errors observed for these variables suggest that residual skewness and heteroskedasticity may have reduced statistical power. Transformations such as log(OOPE + 1) or log(OOPE/total consumption) can stabilize variance but may also introduce interpretational bias and loss of comparability when zeros are present or distributions deviate from log‑normality. Because the primary objective of this cross‑sectional study was to identify statistical associations between financial indicators and psychological distress rather than to model the distributional properties of expenditure data, logistic regression was used as the most appropriate analytic framework. Future research that seeks to model expenditure magnitudes or jointly analyze costs and psychological outcomes analyses should evaluate these transformations or apply generalized linear models with a log link and gamma distribution to assess the robustness of the findings, as recommended in prior methodological literature (65-67). Conventional indicators of financial burden capture only objective expenditures and may neglect the subjective experience of financial toxicity, the perceived cost‑related stress that can independently impair psychological well‑being among cancer survivors and may therefore account for the absence of association observed in this study. Alternatively, following a cancer diagnosis, immediate psychological impact of treatment‑related expenses may be counterbalanced by structural and social buffering mechanisms such as government subsidies, health insurance support, and informal family financing. Moreover, enduring cultural values in Nepal that prioritize resilience, collective responsibility, and acceptance may attenuate the perceived connection between economic hardship and emotional distress, thereby reducing the observable statistical association between financial burden and psychological outcomes. Therefore, the absence of a statistical association should not be interpreted as evidence that financial hardship has no effect on patients’ psychological well‑being; rather, it underscores the need for longitudinal, mixed‑methods research employing context‑sensitive instruments to more accurately examine these complex relationships (68).

The claim that unemployment “increased” after diagnosis is based on retrospective reporting, not longitudinal follow-up. So, causality is not justified.
Recommendations are too general (“Government must strongly implement…”) and not grounded in the study’s findings. More practical, targeted actions (e.g., integrating routine DASS-21 screening in oncology wards, training oncology nurses in basic counseling) need to be mentioned.
**Response: The language describing unemployment trends has been revised (lines 334-346) to remove any causal inference and to reflect the cross‑sectional, retrospective nature of the data.**

The study findings also revealed that psychological distress was notably higher among participants who reported having no occupation after their cancer diagnosis. Lack of a stable income may heighten financial insecurity and psychological dependence, which in turn are associated with reduced self‑esteem and greater emotional distress, consistent with evidence that persistent financial hardship can diminish self‑esteem and contribute to depressive symptoms (51). In this study, the proportion of participants reporting unemployment increased from nearly two-fifths before diagnosis to almost four-fifths after diagnosis, based on retrospective self-reports. However, as this information was not derived from longitudinal follow-up, the observed change should be interpreted as descriptive rather than causal. Future studies employing prospective designs is warranted to examine the reasons for the employment loss after diagnosis, and how it impacts their health and well-being. Prior studies have similarly shown that unemployment and reduced work capacity are common among cancer survivors and are closely linked to psychological distress (52-54).

**The recommendations section (lines 434-447) has also been rewritten to be concise, evidence‑linked, and operationally oriented. It now includes concrete actions, such as integrating routine DASS-21 screening within oncology units and providing focused psychosocial training for oncology nurses.**

These findings underline the need for practical and sustainable interventions to address psychological distress as an integral component of cancer care.

In light of our findings, routine mental-health screening can be incorporated into institutional cancer-care pathways to facilitate early identification and management of psychological distress. Oncology nurses and allied health personnel can be trained in basic psychosocial counselling and communication skills to provide immediate support and facilitate appropriate referrals for both patients and their caregivers, recognizing their shared psychological burden throughout the course of illness.

These actions align with the Nepal Cancer Control Strategy (2024- 2030), which emphasizes enhanced capacity in diagnosis, treatment, and rehabilitation, and the National Health Financing Strategy (2023- 2033), which aims to reduce financial and social vulnerability caused by illness (71, 72). Integrating psychosocial screening and counselling within these national frameworks would help mitigate both the psychological and economic burden of cancer and advance equitable, person‑centered oncology care in Nepal.

Some important limitations are omitted: Cross-sectional design prevents causal inference; possible social desirability bias in face-to-face interviews; use of self-report tool (DASS-21) without clinical validation in this sample; non-response bias from patients who refused/failed to complete DASS-21.
**Response: The limitations section has been expanded (lines 411-429) to acknowledge each of these points explicitly.**

This study has several limitations that should be considered when interpreting the findings. First, the relatively small analytic sample may have reduced statistical power to detect associations and limited the generalizability of results beyond the study sites. The cross‑sectional design restricts causal inference between psychological outcomes and explanatory variables. The use of face‑to‑face interviews may have introduced social desirability bias, as some participants might have under‑reported or modified their emotional experiences to align with perceived expectations of the interviewer. Non‑response and self‑selection bias is also possible, as individuals experiencing greater illness severity or psychological distress may have declined participation owing to fatigue, stigma, or discomfort. Consequently, the reported prevalence of distress may either underestimate or overestimate the true population burden. Second, psychological distress was measured using a self‑reported screening tool rather than clinical evaluation, which may have introduced measurement and cultural response biases. Moreover, the insignificant associations observed between financial hardship indicators (OOPE, CATA, and impoverishment) and psychological distress may partly reflect methodological limitations. Financial hardship is a multidimensional construct that involves material, psychological, and behavioral responses to economic strain, and inconsistencies in its measurement can substantially influence observed relationships (69). In this study, financial data were collected at a single cross‑sectional time point, which may not have coincided with peak treatment phases or periods of acute economic pressure, thereby underestimating the short‑term psychological impact of financial stress (69, 70).

There are recurrent grammar and syntax issues (e.g., “Patients at no cost should undergo through psychological distress” is not correct English). Please consider getting your manuscript reviewed by native English speakers before submitting the revision.
Coherence between sections is uneven: the methods are very detailed, while the discussion feels shallow in parts.
**Response: Revisions has been made. Across the manuscript, we have now:**

- **Refined language, reduced tables, and revised for clarity and academic polish.**
- **Streamlined the Introduction to focus on study rationale and explicit research gaps.**
- **Simplified methodological descriptions while improving transparency regarding sampling, bias, and limitations.**
- **Expanded interpretation to address contextual, structural, and theoretical dimensions.**
- **Strengthened recommendations to ensure policy and practice relevance.**

**In addition,**

Figure file has been processed through the PLOS NAAS tool, and the corrected TIFF version has been included with the revised submission.
