# Supplementary material for: Psychological distress and its associated factors among cancer patients in Nepal: A cross-sectional study
Source: PLOS Ment Health. 2026 Mar 6;3(3):e0000419. doi: 10.1371/journal.pmen.0000419 (PMC12965590; doi:10.1371/journal.pmen.0000419)
Supplement: Attachment — Submitted filename: Response_to_Reviewers_auresp_3.docx [file pmen.0000419.s013.docx]

**12/12/2025**

**Dear Editor and Reviewers,**

We sincerely thank you all for the time and effort spent in evaluating our manuscript. We greatly appreciate the constructive comments and suggestions, which have helped us improve the overall quality of our work.

Below, we provide a point-by-point response to each comment. All changes have been incorporated into the revised manuscript, and relevant line numbers in the updated version are indicated.

Thank you for your insightful and valued contributions!

**Kind regards,**

**Ankit Acharya**

**On behalf of all co-authors.**

**Response to comments**

**Reviewer #1:** Ensure consistency in reporting p-values (e.g., “p = 0.004” vs “p-value = 0.004”). The finding that economic variables (out-of-pocket expenses, catastrophic expenditure) were not significant warrants more discussion- particularly possible confounding or measurement limitations. The conclusion states that psychological distress is linked “primarily to sociodemographic and clinical factors rather than economic burden,” but the discussion does not adequately explain why economic indicators were not significant, given their relevance in LMIC settings. Consider comparing findings to regional or global studies to contextualize whether these levels are expected.

**Response: We thank the reviewer for noticing this detail. All p‑values have been standardized throughout the manuscript as “p-value = …”, and the entire manuscript has been reviewed to ensure consistency in statistical reporting.**

**Additional explanation has been incorporated in the Discussion (in the lines 402-428) to address possible confounding, measurement limitations, and contextual factors that may account for the non-significant association between economic indicators and psychological distress, with reference to relevant regional and global literatures. We believe these revisions clarify our interpretation and strengthen the conceptual alignment between the results, discussion, and conclusion.**

**Revised section:**

**Unmeasured variables such as coping capacity, resilience, health literacy, household debt, and pre‑existing mental‑health conditions represent additional measurement constraints, as their exclusion may have introduced residual confounding by influencing both financial hardship and psychological distress.**

**Beyond analytic considerations, real‑world contextual factors may also shape the association between financial hardship and psychological distress. Following a cancer diagnosis, the immediate psychological impact of treatment‑related expenses may be counterbalanced by structural and social support mechanisms such as government subsidies, health insurance, and informal family financing, particularly in developing countries where collective responsibility, extended‑family support, and informal financial networks are central to illness management, and where social connectedness has been shown to foster resilience and emotional recovery among cancer survivors (67-70). The literature indicates that financial strain, emotional distress, and social and spiritual disruption constitute a broader “psychosocial cost burden” experienced by patients and families (5, 71). Jones and colleagues (2024) argue that multiple moderating factors including socioeconomic status, self‑efficacy, and social support can either amplify or attenuate distress (72). Similarly, Park and Look (2018) observed that although higher financial burden showed a significant association with poorer quality of life and greater psychological distress among cancer patients, its association with depressive symptoms was not significant after adjusting for covariates. This finding suggests that depressive symptoms do not always correspond to the magnitude of financial pressure, possibly because of the already high baseline level of psychological distress (73) or the buffering effect of protective sociocultural influences. These observations imply that even when treatment costs are high, their psychological impact may be moderated or masked by strong structural and social support mechanisms. The absence of a statistical association in this study should therefore not be interpreted as evidence that financial hardship has no effect on patients’ psychological well‑being, but as a call for longitudinal, mixed‑methods research using context‑sensitive instruments to examine these relationships rigorously.**

**Reviewer #3:**

Have the authors made all data underlying the findings in their manuscript fully available (please refer to the Data Availability Statement at the start of the manuscript PDF file)?

Reviewer #3: No

**Response: All data underlying the findings are fully available. The complete dataset and summary results are included within the manuscript and its Supporting Information files (see S1 Dataset).**

**Additional revisions:**

- Line 385: Revision grammar from “Contrary to our expectations…” to “Contrary to expectation…)
- Line 392: Removed the term “statistical” as it was implicitly understood.
- Line 396: Removed the redundant term “analyses”.
- Line 424-428: Improved grammatical structure and clarity.
- Relevant literatures have been cited.
- The *citation “Roshid MM, Rahman MM, Sarker MH, Rahman MJ, Prihanto JB, Alam MN, et al. Assessment of psychological distress and its associated factors among patients with cancer undergoing chemotherapy in rural Bangladesh. Journal of Family Medicine and Primary Care. 2024;13(2):647-55.”* was listed twice, and has now been retained only once in the manuscript and revised reference list.
