# Supplementary material for: Psychological distress and its associated factors among cancer patients in Nepal: A cross-sectional study
Source: PLOS Ment Health. 2026 Mar 6;3(3):e0000419. doi: 10.1371/journal.pmen.0000419 (PMC12965590; doi:10.1371/journal.pmen.0000419)
Supplement: Attachment — Submitted filename: Response_to_Reviewers_auresp_4.docx [file pmen.0000419.s014.docx]

**03/01/2026**

**Dear Editor and Reviewers,**

We sincerely thank you all for the time and effort spent in evaluating our manuscript. We greatly appreciate the constructive comments and suggestions, which have helped us improve the overall quality of our work.

Below, we provide a point-by-point response to each comment. All changes have been incorporated into the revised manuscript, and relevant line numbers in the updated version are indicated.

Thank you for your insightful and valued contributions!

**Kind regards,**

**Ankit Acharya**

**On behalf of all co-authors.**

**Response to comments**

**Reviewer #1:**

1. Still needing stronger theoretical or conceptual framework.

**Response: We strengthened the theoretical and conceptual framework in the Introduction by expanding the discussion of contemporary models linking socio-demographic, clinical, and economic factors to psychological distress (Lines 86-114).**

2. Provide logical explanation on what guided the selection of sociodemographic, clinical, and economic variables. The study was conducted in two tertiary hospitals, which may overrepresent advanced-stage cancer cases and economically vulnerable patients, including rural or non-tertiary care settings.

**Response: We added a new subsection, Selection of Study Variables, to the Methods section (Lines 199-217) explaining the rationale for including these variables. We also revised the study setting description (Lines 141-147) and the Limitations section (Lines 489-494) to acknowledge that tertiary referral hospitals may overrepresent patients with advanced disease and higher financial burden.**

3. Reconcile this: DASS-21 measures symptom severity, not clinical diagnoses and high prevalence rates (>65%) raise the possibility of measurement inflation due to somatic overlap with cancer symptoms. Discuss the possibility that cancer-related physical symptoms may artificially elevate DASS-21 scores.

**Response: We clarified in the Discussion (Lines 364-372) that the DASS-21 assesses symptom severity rather than clinical diagnosis and discussed the potential inflation of scores due to overlap with cancer-related somatic symptoms.**

4. Consider cultural norms, financial disclosure or family support systems to explain the lack of observed association.

**Response: We expanded the Discussion (Lines 473-477) to incorporate cultural norms, financial disclosure practices, and family/community support networks as possible explanations for the lack of observed association.**

We are grateful to Dr. Gino A. Cabrera for their thoughtful review and valuable suggestions.
